# Mediation of Ethnic Disparity in the 5-Year Mortality of Cervical Cancer Patients in the US, 2001–2019

**DOI:** 10.3390/healthcare13090964

**Published:** 2025-04-22

**Authors:** Shi-Hao Zhou, Yong-Qiao He, Hua Diao, Da-Wei Yang, Tong-Min Wang, Ying Liao, Wei-Hua Jia, Wen-Qiong Xue

**Affiliations:** 1 State Key Laboratory of Oncology in South China, Collaborative Innovation Center for Cancer Medicine, Guangdong Key Laboratory of Nasopharyngeal Carcinoma Diagnosis and Therapy, Sun Yat-sen University Cancer Center, Guangzhou 510060, China; zhoushh33@mail2.sysu.edu.cn (S.-H.Z.); heyq@sysucc.org.cn (Y.-Q.H.); wangtm@sysucc.org.cn (T.-M.W.); liaoying@sysucc.org.cn (Y.L.); 2 School of Public Health, Sun Yat-sen University, Guangzhou 510080, China; 3 Department of Gastroenterology, Third Military Medical University Xinqiao Hospital No.83, Xinqiao Street, Shapingba District, Chongqing 400037, China; diaoh@mail2.sysu.edu.cn; 4 Guangdong Provincial Center for Disease Control and Prevention, 160 Qunxian Road, Dashi Street, Panyu District, Guangzhou 511430, China; yangdw3@mail2.sysu.edu.cn

**Keywords:** cervical cancer, ethnic inequity, mortality, Surveillance, Epidemiology, and End Results (SEER)

## Abstract

**Objectives:** This study aims to investigate the potential mediators for ethnic disparity in cervical cancer 5-year mortality and identify potential patients affected by ethnic disparities. **Methods:** The cohort study analyzed 56,374 cervical cancer patients from the Surveillance, Epidemiology, and End Results (SEER) 17 database (2000–2019). The primary and secondary outcome were the 5-year mortality of cervical cancer patients for all causes and cervical cancer-specific death, respectively. Cox and competing risks models were applied to identifying prognostic factors for 5-year cervical cancer all-cause mortality and specific death, respectively. Potential mediators for ethnic disparity were analyzed using multiple mediation analyses. **Results:** NHB patients had a 49% higher risk of 5-year mortality than NHW patients, while Hispanic and API patients showed a 19% and 12% decreased risk, respectively. Mediation analyses revealed that clinical stage and surgery predominately contributed to NHW-NHB prognosis disparities, with an indirect effect proportion of 29.6% and 26.7% for all-cause mortality and 34.2% and 26.7% for disease-specific death, respectively. No significant mediation effect was observed for other ethnic disparities. Compared to NHW patients, the inferior prognosis of NHB patients was observed mainly for localized and regional cancer, receiving hysterectomy, and, especially, adenocarcinomas. Conversely, the superior prognosis of Hispanic and API patients was observed in the no surgery subgroup and mainly for squamous cell carcinomas. **Conclusions:** Delayed diagnosis and a lack of surgery are key contributors to the prognosis discrepancy between NHB and NHW patients. More attention should be paid to NHB patients with cervical adenocarcinoma to narrow the disparity.

## 1. Introduction

Nowadays, the ethnic disparity for cancer incidence and outcomes has been well documented [1,2], especially for the most common cancers such as lung cancer [3], breast cancer, prostate cancer [4,5], and so on. Biological factors including molecular subtypes, oncovirus infection and other factors may contribute to this disparity [6,7]. Meanwhile, socioeconomic factors more profoundly and comprehensively affect cancer outcomes through health consciousness, individual behavior, screening approaches, and treatment received [7,8,9], which construct a complex network. To disentangle the effect size of these factors for certain cancers would contribute to prioritizing strategies or interventions for eliminating the ethnic inequities in cancer healthcare [7].

Cervical cancer has become the most likely preventable and curable cancer because of vaccines for human papillomavirus (HPV) and screening strategies [10]. However, there is still a big gap between current incidence and the target of the Cervical Cancer Elimination Initiative launched by the WHO (<4/100,000 person-years) [11]. In the US, the incidence and mortality remained relatively stable after 2012 [12] and even increased for certain age and ethnic subgroups [13,14], posing a significant and long-standing challenge for the management and treatment of cervical cancer patients. Substantial ethnic disparity has been reported for the prognosis of cervical cancer [15,16], with age-adjusted mortality almost two times higher in black people than in the white people (5.0 vs. 2.6 per 100,000 person-years) [17]. Two recent studies provide clues about the factors affecting these ethnic disparities [17,18]. Insurance status may act as a primary mediator (proportion: 51.3%) for disparity in the early detection of cervical cancers between white and black women [18]. Further, black women have higher mortality compared to white women, especially in advanced stages of cervical adenocarcinomas [17]. This evidence suggests that both socioeconomic and histological factors play roles in ethnic disparities. The significant association between ethnicities and cervical cancer mortality remained after adjustment for clinical stages, histological types, and other confounding factors [19], indicating other factors may also play roles in the disparity. It is urgently necessary to decompose the effects of these factors for cervical cancer mortality so as to identify potential modifiable factors affecting the outcomes. Nevertheless, few studies have simultaneously examined the impacts of socioeconomic and biomedical factors on ethnic disparities in cervical cancer mortality. How and to what extent these factors influence the disparities remains to be studied. In addition, exploring subgroups with more pronounced ethnic disparity may also provide clues for tailored inventions to improve survivability.

Accordingly, we conducted this analysis using data from the Surveillance, Epidemiology, and End Results (SEER) database, aiming to identify primary factors underling ethnic disparity in cervical cancer prognosis. We assessed the associations between ethnicities, as well as multiple sociodemographic and clinical factors, with total and cervical cancer-specific 5-year mortalities, to test whether they were independent risk factors. We then identified potential mediators of the disparities and assessed their contribution via mediation analysis. Moreover, we conducted multilevel subgroup analysis to identify the subpopulations who experienced more pronounced ethnic disparities as a target for further attention or tailored strategies to eliminate healthcare inequities in the management of cervical cancer.

## 2. Methods

### 2.1. Data Source and Study Population

We performed a population-based study using data from the US National Cancer Institute’s SEER program, which cover almost one-third of the populations in the United States [20]. The SEER databases contain individual information including demographic, socio-economic status, histology, stage, treatment, and survival data of large cancer patient samples from cancer registries in the US, thus allowing comprehensive analyses of prognostic factors. The cancer diagnoses were defined using the International Classification of Diseases for Oncology, Third Edition (ICD-O-3), ranging from C53.0–C53.9. This study followed the reporting guidelines of the Strengthening the Reporting of Observational Epidemiological Studies (STROBE) statement.

We extracted the records of all women (*N* = 67,082) diagnosed with cervical cancer between 2000 and 2019 from the SEER database (17 registries, submitted in November 2021) through SEER*Stat software (Version 8.4.2). We then excluded 4461 patients diagnosed with secondary cancer and 5150 patients with multiple cancers. Secondary cancer refers to malignancies diagnosed at a site distinct from the original (primary) cervical cancer, including metastatic or recurrent cancers. This exclusion aims to minimize confounding effects from biologically distinct disease processes. Forty patients aged under 18 years and 3 patients with in situ cancer were also excluded. We excluded 369 patients with their diagnostic information obtained only from death certificates or autopsies to avoid potential misclassification. In addition, patients lacking ethnic information were excluded. Finally, a total of 56,374 patients were included in the analyses.

### 2.2. Study Variables and Outcomes

We retrieved all available information on demographics (age, ethnicity, state, and year of diagnosis), socio-economic factors (marital status, income, and rurality), diagnoses (stage, histologic subtypes, and grade), treatments (surgery, radiation therapy, and chemotherapy), and survival data for each patient from the SEER database. For the primary exposure variable of ethnicity, all patients were grouped into five mutually exclusive categories: non-Hispanic white (NHW), non-Hispanic black (NHB), Hispanic (all races), non-Hispanic Asian/Pacific islander (API), and non-Hispanic American Indian/Alaska Native (AI/AN) [21]. Socio-economics factors (e.g., marital status, income, rurality) may influence healthcare accessibility and treatment opportunities, affecting cancer prognosis. And the clinical factors (e.g., stage, histologic subtype, grade, surgery, radiation therapy, chemotherapy) can directly affect cancer prognosis. These factors may collectively contribute to prognostic disparities. Given their likely roles in cancer prognosis, we included these variables as potential effect modifiers in the Cox proportional hazards model. Those with statistical significance in the Cox regression model were further treated as potential mediators for subsequent mediation analysis. The diagnostic time was divided into four periods (2000–2004, 2005–2009, 2010–2014, and 2015–2019) based on the year of diagnosis. And the regions were defined based on the states where the cancer registries were located. Three variables represent socio-economic characteristics, including marital status (married or unmarried), income levels (less than $50,000/year, $50,000-$60,000/year, $60,000-$70,000/year, or more than $70,000/year), and rurality (based on the rural–urban continuum code developed by the United States Department of Agriculture [22]: metropolitan counties with a population of more than 1 million, 250,000 to 1 million, or fewer than 250,000 and nonmetropolitan areas). For clinical factors, six variables were included: stage (localized, regional, or distant), histologic subtype (squamous cell carcinoma, adenocarcinoma, and other types), pathological grade (I to IV), surgery (yes or no), radiation therapy (yes or no/unknow) and chemotherapy (yes or no/unknow). Except for radiation therapy and chemotherapy, the missing values for other variables were categorized on a single level.

The determination of death in the SEER database is based on information uploaded by the registries, including death certificate materials [23]. The primary outcome of interest was a 5-year mortality of all causes for cervical cancer patients. The survival time was defined as the period between diagnosis and death from any cause. Patients were diagnosed between 2000 and 2019 and were followed up until 31 December 2019. Furthermore, we restricted the analysis to cervical cancer-specific death according to the SEER death classification code [24].

### 2.3. Statistical Analysis

Firstly, the demographic, socioeconomic, and clinic characteristics of patients were described and compared among ethnicities using the Pearson χ^2^ test for categorical variables and one-way ANOVA analysis for age. To explore the potential heterogeneity of ethnic disparity, cumulative incidence curves of cervical cancer-specific causes and other causes for different ethnicities were plotted by demographic, socioeconomic, and diagnostic subgroups, based on the competing risk estimates from Fine and Gray competing risk proportional hazard models [25].

Then, to explore potential prognostic factors for cervical cancer, we calculated the hazard ratio (HR) and 95% confidence interval (95% CI) of 5-year mortality for all factors, in both the univariate model and multivariate model, adjusted by all other factors, using a Cox proportional hazard regression model. Due to the presence of competing risks (e.g., non-cancer deaths), Fine and Gray competing risk proportional hazard models [26] were selected to ensures unbiased estimation of cancer-specific mortality. We conducted this model to generate HRs of cervical cancer-specific mortality adjusted by other factors, and deaths from all causes other than cervical cancer were treated as competing events. Moreover, taking the NHW group as a reference, we calculated adjusted HRs with all demographic, socioeconomic, and clinic characteristics added one by one in order to find the predominant mediators.

We further conducted multiple mediation analyses to decompose the effect of these factors influenced by ethnicities that affect the disparity of cervical cancer prognosis (Figure S1). Multiple mediation analysis, based on the counterfactual framework, extends traditional methods to consider multiple mediators or variables of different types simultaneously. It can isolate each mediator’s indirect effect for comparison to assess importance and avoid traditional sequential assumptions. Using generalized models with multiple additive regression trees, the analyses can integrate multilevel variables and can better account for potential interaction and complicated predictor–mediator effects [8,27]. Two variables, age and year of diagnosis, were treated as confounding factors and adjusted in all models. All other factors significantly associated with 5-year mortality (marital status, income, stage, histologic subtype, grade, surgery, radiation therapy, and chemotherapy) were regarded as potential explanations for the ethnic disparity. Relative effect sizes for each potential mediator, total, indirect, and direct, were reported as percentages to show their contributions to prognosis of cervical cancer patients.

In the end, we also performed association analyses between major ethnicities and 5-year cervical cancer prognosis in subgroups of primary mediators, using Cox regression models and Fine and Gray models, to identify target subgroup patients most affected by ethnic disparity.

All analyses were performed using R, version 4.2.0 (R Group for Statistical Computing). The prognostic analyses were conducted using the survival [28] (Cox regression model) and cmprsk [26] (Fine and Gray model) package. The mediation analyses were conducted using the mma [29] package. The results were visualized using the ggplot2 and survminer package. All statistical tests were two-sided, and a *p*-value of less than 0.05 was considered to indicate statistically significant differences.

## 3. Results

### 3.1. Patient Characteristics and Outcomes

A total of 56,374 women diagnosed with primary cervical cancer between 2000 and 2019 from the SEER 17 database were included in the analysis. Half of the participants were categories as NHW (52.3%, n = 29,473); 24.3% as Hispanic (n = 13,691), 12.9% as NHB (n = 7273), 9.7% as API (n = 5450), and 0.9% as AI/AN (n = 487). The median ages for these ethnicities were 47, 45, 50, 50, and 44 years, respectively. The proportions for patients diagnosed with localized cancer were 47.5%, 46.8%, 36.5%, 44.1%, and 45.0%, respectively. Squamous cell carcinomas (SCCs) were the predominant histologic subtype for all ethnicities, with percentages of 63.5%, 68.2%, 75.5%, 64.8%, and 68.2%, respectively. The distribution of other demographic, socioeconomic, and treatment characteristics among different ethnicities is displayed in Table 1. During the follow-up period, about one-third of the patients died (36.3%, n = 20,462). Most of these deaths occurred in the first five years after diagnosis, with a cumulative death rate of 31.0%. Cervical cancer-related death was the predominate cause, with a 5-year cumulative death rate of 26.5% (Table S1).

### 3.2. Association Between Ethnicities and 5-Year Mortality of Cervical Cancer

Marked disparities of 5-year mortality were observed among ethnic groups; NHB patients had the highest 5-years cumulative death rate of 42.3% for all causes and 35.5% for disease-specific deaths, followed by NHW and AI/AN patients with 31.6% and 32.7% for all-cause death and 27.2% and 27.9% for disease-specific death, respectively. Hispanics and API patients had the lowest 5-year cumulative death rates of 24.8% and 27.6% for all-cause death and 21.4% and 23.6% for disease-specific death (Table S1). The results from competing risk models were consistent, with the NHB group having persistently higher death rates for cervical cancer-specific causes through the entire 5-year follow-up compared to other groups; NHW and AI/AN patients ranked lower than NHB patients, but higher than the Hispanic and API group (Figure S2). In addition, subgroup analyses by all sociodemographic factors found similar results. NHB patients had the poorest prognosis among all subgroups (Figures S3–S8). Although the 5-year mortality risk for patients with different grades, stages, and histological subtypes varied dramatically, NHB patients remained the worst-performing group in all subgroups (Figure S9–S11).

NHB patients showed a 49% higher risk of 5-year mortality without adjustment, while the Hispanic and API groups had a 19% and 12% decreased risk of mortality compared to NHW patients. The 5-year mortality risks for AI/AN patients were not significantly different from the NHW group, which may be due to insufficient sample size. After adjusting for potential confounding factors including age, year of diagnosis, marital status, income level, rurality, region, and medical conditions, NHB patients still showed a significantly higher risk of 5-year mortality, while their HR attenuated to 1.09 (95% CI, 1.05–1.14). The mortality risks for Hispanic and API patients remained significant, with adjusted HRs of 0.83 (95%CI, 0.80–0.87) and 0.80 (95%CI, 0.76–0.85), respectively. HRs for 5-year cervical cancer-specific mortality were equivalent to the estimates for all-cause mortality (Table S2).

### 3.3. Mediation of Ethnic Disparity in the 5-Year Mortality of Cervical Cancer

**Figure 1:**
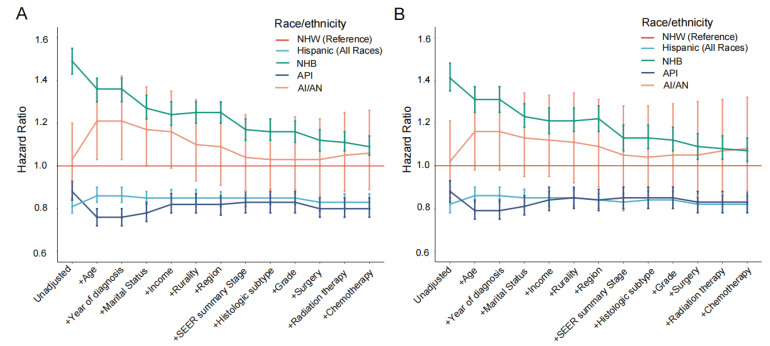
Ethnicity hazard ratios for 5-year mortality of all causes (**A**) and cervical cancer-specific causes (**B**). The bars denote 95% CI for the corresponding hazard ratios. HRs for 5-year overall mortality were calculated using Cox regression models adjusted by all other variates, including ethnicity, age, marital status, years of diagnosis, income, rural–urban continuum code, region, SEER summary stage, histologic subtype, grade, radiation therapy, chemotherapy, and surgery. HRs for 5-year cervical cancer-specific mortality were calculated using Fine–Gray competing risk models adjusted by all other variates, including ethnicity, age, marital status, years of diagnosis, income, rural–urban continuum code, region, SEER summary stage, histologic subtype, grade, radiation therapy, chemotherapy, and surgery. Abbreviations: AI/AN, American Indian/Alaska Native; API, Asian or Pacific Islander; NHB, non-Hispanic black; NHW, non-Hispanic white.

Multivariate analysis adjusted for all other factors revealed that all demographic, socioeconomic, and clinical factors except rurality were independently associated with a 5-year mortality risk of cervical cancers (Table S2). Furthermore, when we added these 12 factors one by one into the regression models, adjusted HRs for NHB patients decreased gradually (Figure 1 and Table S3). Marital status and clinical stages were the most significant adjustments compared to other factors. HRs for the Hispanic and API patients were not substantially changed after adjustment.

**Figure 2:**
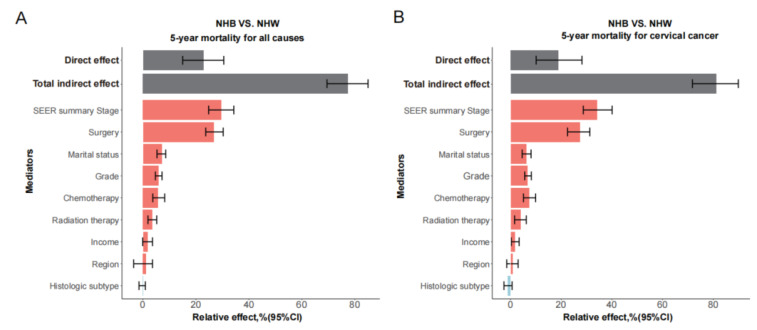
Mediation analysis for the ethnic disparity in patients with cervical cancer. Total indirect effect refers to the combined mediation effect through all mediators, while Direct effect reflects the effect of the exposure on the outcome that is not explained by the included mediators (negative mediation, blue; positive mediation, red).

Subsequently, we conducted multiple mediation analyses with all potential mediators to assess the contributions of these factors to the ethnic disparity of cervical cancer mortality. For the disparity between NHW and NHB patients, about four fifth (77.2%; 95%CI, 69.5–85.0%) of the effect were attributed to these mediators. The clinical stages and surgery (yes/no) explained 29.6% (95%CI, 24.8–34.3%) and 26.7% (95%CI, 23.7–30.3%) of the total effect, respectively (Table S4), far higher than other mediators. Marital status, grade, radiation therapy, and chemotherapy all contributed significantly to disparity, with a proportion of 7.1%, 5.9%, 3.5%, and 5.6%, respectively. The corresponding relative effect was higher for cervical cancer-specific mortality, with 34.2% for stage and 27.3% for surgery (Figure 2 and Table S4), while a similar result was observed in subgroup analysis by income (Table S5). The overall mediation effect for disparities between API and NHW patients was not significant. Nevertheless, income levels and marital status accounted for 13.3% (95%CI, 2.6–23.3%) and 8.6% (95%CI, 4.7–12.5%) of disparity between API and NHW patients, while clinical stages and grades contributed to inferior prognosis among API patients, which may counteract the previous prognostic advantage. For disparity between Hispanic and NHW patients, histologic subtypes showed a relative effect of 5.2%, but with a null overall indirect effect (Table S4). The mediators and mediation effects for disease specific 5-year mortality were essentially the same or slightly higher than the overall survival.

### 3.4. Subgroup Analysis by Clinical Stages, Histologic Subtype and Treatment

**Figure 3:**
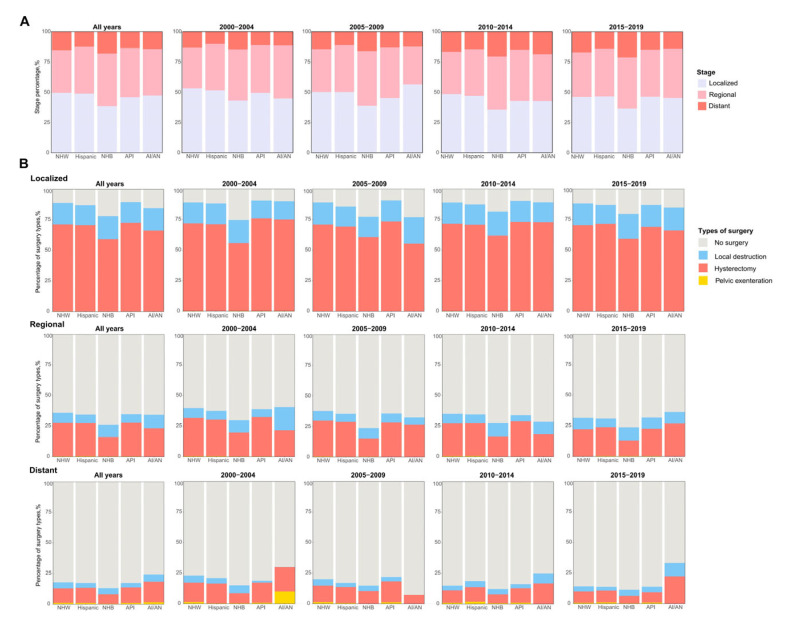
The proportions of clinical stages and surgery types by stage and ethnicity during different periods. (**A**) The proportions of clinical stages by ethnicity during different periods. (**B**) The proportion of surgical types among different ethnic groups stratified by clinical stage and different periods. Abbreviations: AI/AN, American Indian/Alaska Native; API, Asian or Pacific Islander; NHB, non-Hispanic black; NHW, non-Hispanic white.

Consistently with the results of mediation analysis, NHB patients had lower rates of localized cancers but higher rates of distant cancers compared to other ethnic groups. This trend persisted throughout the 20-year period (Figure 3A). In addition, NHB patients had lower rates of receiving hysterectomies than NHW patients through the time period, especially for localized and regional cancers. The proportions of Hispanic and API patients receiving hysterectomies were similar to that of NHW patients (Figure 3B).

**Figure 4:**
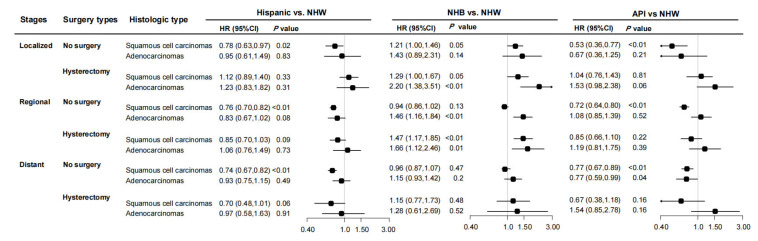
The association between ethnicities and 5-year mortality among cervical cancer patients by stages and histologic types. Abbreviations: AI/AN, American Indian/Alaska Native; API, Asian or Pacific Islander; CI, confidence interval; HR, hazard ratio; NHB, non-Hispanic black; NHW, non-Hispanic white.

Since surgical decisions were highly related to clinical stage, we analyzed the association between ethnic groups and 5-year mortality through stage–surgery subgroups. Intriguingly, disparity varied dramatically across subgroups. NHB patients had a significantly poorer prognosis (adjusted HR = 1.39) than NHW patients in localized and regional cases receiving hysterectomy, while not in distant cancer. On the contrary, Hispanic and API patients had lower mortality mainly in no surgery subgroups for all stages (Figure S12). When the patients were divided by histologic subtypes and stages, the disparity between NHB and NHW patients was remarkable in adenocarcinoma (HR = 1.41; 95%CI, 1.25–1.58), as well as in localized SCC (HR = 1.35; 95%CI, 1.17–1.55). However, disparity between Hispanic and API patients and NHW patients was significant mainly for SCC, while not for adenocarcinomas (Figure S13). Benefiting from the large sample size of SEER data, we conducted the analyses grouped by stage, surgery, and histologic subtype simultaneously. A remarkably higher risk of 5-year mortality was observed in NHB patients with localized adenocarcinoma and receiving hysterectomy (HR = 2.20; 95%CI, 1.38–3.51), as well as in reginal adenocarcinomas (no surgery: HR = 1.46, 95%CI = 1.16–1.84; hysterectomy: HR = 1.66, 95%CI, 1.12–2.46) and regional SCC receiving hysterectomy (HR = 1.47, 95%CI = 1.17, 1.85), whereas a significantly lower risk of mortality was observed in Hispanic and API patients with SCC patients receiving no surgery, especially in SCC. HRs were within the ranges of 0.74–0.78 and 0.53–0.77 for Hispanic and API patients, respectively (Figure 4). The associations between ethnicities and 5-year cervical cancer-specific mortality were similar to the results for total mortality (Tables S6–S8).

## 4. Discussion

Using nationwide data from the SEER program, our study identified the potential mediators for ethnic disparity in the mortality of cervical cancer and quantified their effects. Consistently, NHB patients had a notably 49% higher 5-year mortality for all-cause and cervical-cancer specific death compared to NHW patients, while Hispanic and API patients had 12–19% lower 5-year mortalities than NHW patients. After adjusting for all available potential confounders including socioeconomic factors and clinical factors, NHB women exhibited a 9–10% higher 5-year mortality risk compared to NHW patients, similar to previous studies [30,31]. Given the large NHB population and high incidence of cervical cancer among NHB women, a 9% excess mortality risk translates into a substantial disease burden. A recent study estimated that there may be 2180 new cervical cancer cases and 610 cervical cancer deaths among NHB women in 2025, underscoring its public health importance [32]. Apart from the factor of clinical stages, which is widely known [18], we propose that undergoing surgery is the second most important prognostic factor (a relative effect of 26.7% for all-cause death and 27.3% for cervical cancer-specific death), mediating the ethnic disparity between NHB and NHW patients. Notably, stratified analysis specified that early-stage patients, especially with adenocarcinoma and receiving hysterectomy, exhibited more profound NHB–NHW disparity after adjusting for potential confounding factors, while Hispanic and API patients showed significantly lower mortality, mainly in the no surgery group. These results suggest that modifiable factors related to screening and surgery could have an impact on cervical cancer prognosis, and tailored strategies need to be conducted to eliminate ethnic and ethnic inequity.

A remarkable disparity in cervical cancer survivability has been observed between the white and the black population throughout the last few decades [16,33]. Efforts have been made to explore reasons and potential modifiable factors that may narrow this disparity [31,34,35]. It has been found that black women tend to be diagnosed with late-stage cervical cancer, thus having inferior prognoses. Other factors including age, year of diagnosis, and histological subtypes could also affect ethnicity-differentiated prognoses [34]. However, the extent to which clinical stage modulated disparity and the potential involvement of other factors were largely undefined. Consistent ethnic disparity based on different socioeconomic levels (e.g., income, rurality) indicated that multiple complex factors contribute to disparity. Our study corroborated that clinical stage is the most important mediator for the disparity between NHB and NHW patients, accounting for about 30% of disparity. And the disparity regarding early diagnosis between NHB and NHW patients has not noticeably improved since 2000. A recent study further explored the mediators of these ethnic inequities [18] and found that half of the stage-related inequities were mediated by insurance status. Similarly, lower socioeconomic levels were associated with the diagnosis of delayed-stage cervical cancer, especially in NHB women [36]. Screening is the most effective way to raise the early diagnostic rate for cancers. A new study using population-based data found that the cervical cancer screening rate was concurrent with the survivability of patients, with NHB patients having the lowest rate of 53.2% and Hispanics, API, and NHW patients having 65.4%, 66.5%, and 63.5%, respectively [37]. This disparity may be due to the lack of community education and awareness of cervical cancer and its intervention strategies [35], suggesting that future policy and campaigns should focus more on NHB people.

Noteworthily, our results revealed that undergoing surgery is the second most important mediator of ethnic disparity in 5-year mortality between NHW and NHB patients, explaining more than one-fourth of the disparity. It was shown that NHB patients had a lower rate of receiving hysterectomy than NHW patients, for all stages and throughout the 20-year period. This result is in line with previous studies [18,38]. Subgroup analysis by stage showed that ethnic disparity was significant only in localized cancer and not in distant cancer, also indicating a deficiency in the provision of surgeries for NHB patients, since surgery is a more effective therapy for early-stage cervical cancer than distant cancer. In addition, the NHB–NHW disparity was more significant in early-stage patients receiving hysterectomy than those receiving no surgery or local destruction. Although the impact of surgical treatments on ethnic disparities in cervical cancer prognosis has drawn little attention, evidence involving women with benign gynecologic diseases and undergoing hysterectomy found that black women tended to receive open hysterectomy and have more postoperative complications than white women [39,40]. Investigations among patients with other diseases observed that black patients tended to undergo surgery at low-quality hospitals [41] and had a higher rate of 30-day mortality than white patients [42]. It is reasonable to assume that low-quality hysterectomy surgery contributed to NHB–NHW disparity in cervical cancer prognosis. Further, local destruction could be a viable alternative to hysterectomy for NHB patients as no significant NHB–NHW disparity was observed in patients receiving this therapy, consistent with a recent study for early-stage cervical cancer [43]. These results highly suggested that NHB patients should opt more actively for surgical treatment and cautiously select the surgery procedure based on medical conditions. Conversely, the Hispanic and API patients had superior survivability compared to NHW patients in the no surgery group, but not in those undergoing hysterectomies. This phenomenon could be partly explained by healthier lifestyle choices, such as low-fat diets, less smoking, and lower alcohol intake [44], or superior mental health [45] among Hispanic and Asian populations. However, further research is still required to specify potential reasons.

Moreover, we observed that the NHB–NHW disparity mainly occurred in adenocarcinoma and localized SCC, while the Hispanic–NHW and API–NHW disparity mainly occurred in SCC. To our knowledge, the ethnic disparity of cervical cancer was first observed to vary between histological subtypes in fully adjusted models. Although previous studies have explored ethnic disparities in cervical cancer prognosis and identified some factors influencing these disparities, such as socioeconomic indicators and insurance status, most of these studies have not focused on prognostic disparities across histological subtypes [31,46,47,48]. In our study, we systematically investigated and quantified the mediators contributing to these ethnic disparities. We found that clinical stage and surgery were the most influential mediators accounting for NHB–NHW disparities. Furthermore, we also observed that ethnic disparities vary across histological types, stages, and surgery methods and were most pronounced in localized cancer. Previous studies have demonstrated that cervical cancer adenocarcinoma and SCC have differentiated genetic profiling and immune features [49,50], suggesting biological factors may play roles in their prognosis. NHB patients with localized adenocarcinoma showed a remarkably inferior prognosis compared to NHW patients, indicating the necessity of multiple adjunctive therapies or systematic therapy targeting this subtype. Additionally, consistent evidence demonstrates that cervical adenocarcinoma exhibits significantly poorer prognosis and elevated recurrence rates compared to squamous cell carcinoma (SCC) [51,52], probably necessitating proactive follow-up treatment management. In our study, NHW–NHB prognosis disparity is more profound in patients with adenocarcinoma and among patients who underwent hysterectomy. (Figure S12, Tables S6 and S7). These findings highlight the importance of histology-specific management approaches in NHB patients, underscoring the need for high-quality cancer treatment and standardized surveillance protocols in adenocarcinoma patients.

Benefiting from the population-based large sample-size of SEER data, we were able to conduct comprehensive overall analysis and specific stratification analysis for various factors to identify prognostic factors influencing ethnic disparities. However, several limitations should be considered. Firstly, since it was not available in SEER data, we did not include insurance information, which may have a more direct impact on the availability of and accessibility to healthcare. Nevertheless, we used individual income level as a representative factor for total socioeconomic status, which was positively related to health insurance [53]. In addition, a recent study reported that insurance and other socioeconomic factors were more likely to play indirect roles in the access to screening and treatment of cancers, rather than having a direct impact [18,54], which supports our results. Secondly, emerging treatments including target therapy and immunotherapy, which have pivotal impacts on prognosis, were not included in the analysis. However, these treatments were mainly used for a portion of patients with late-stage cervical cancer [55], which did not show apparent ethnic disparities. Moreover, adjuvant radiation and chemotherapy were adjusted overall and in each subgroup analysis, providing reliable results. Thirdly, in our mediation analysis, surgery and clinical stage were identified as primary mediators of NHB–NHW ethnic prognosis disparities. However, potential unmeasured confounders, such as hospital quality and implicit bias in clinical decision-making, may jointly influence both mediators (e.g., delayed surgery) and clinical outcomes. While we adjusted for available confounders and employed multivariable mediation analyses to isolate the independent effects of each mediator, residual confounding remains possible. Future studies should incorporate institutional-level data (e.g., hospital accreditation status) and implicit bias metrics to further elucidate the underlying mechanisms driving disparities in surgical access and outcomes.

## 5. Conclusions

In summary, using population-based SEER data, we disentangled the ethnic disparity of cervical cancer prognosis and highlighted the effects of both early diagnosis and surgery treatment on the 5-year mortality of cervical cancer. An enduring disparity of early diagnosis was observed between NHB and NHW patients, which supports the demand for more community-based education programs to improve knowledge and screening behaviors in the NHB population. In addition, it is noticeable that the lack of proper surgeries may lead to lower survivability in NHB patients, accounting for nearly one-third of NHB–NHW disparity. And tailored treatment strategies should be applied for NHB patients, especially for the histological type of adenocarcinoma.

## Figures and Tables

**Table 1:** Patient demographic, socioeconomic, and clinical characteristics by ethnicity.

	Ethnicity						
Characteristics	Overall (n = 56,374), N (%)	Non-Hispanic White (n = 29,473), N (%)	Hispanic (All Races) (n = 13,691), N (%)	Non-Hispanic Black (n = 7273), N (%)	Asian or Pacific Islander (n = 5450), N (%)	American Indian/Alaska Native (n = 487), N (%)	*p* Value
Age, median (IQR)	47 (38–59)	47 (38–59)	45 (37–56)	50 (40–62)	50 (42–62)	44 (35–55)	<0.001
Year of diagnosis							<0.001
2000–2004	14,201 (25.2)	7641 (25.9)	3356 (24.5)	1892 (26.0)	1216 (22.3)	96 (19.7)	
2005–2009	13,916 (24.7)	7374 (25.0)	3370 (24.6)	1763 (24.2)	1290 (23.7)	119 (24.4)	
2010–2014	13,615 (24.2)	7097 (24.1)	3253 (23.8)	1755 (24.1)	1376 (25.2)	134 (27.5)	
2015–2019	14,642 (25.9)	7361 (25.0)	3712 (27.1)	1863 (25.6)	1568 (28.8)	138 (28.3)	
Marital Status							<0.001
Married	23,858 (42.3)	13,409 (45.5)	5608 (41.0)	1684 (23.2)	3005 (55.1)	152 (31.2)	
Unmarried	29,183 (51.8)	14,256 (48.4)	7337 (53.6)	5125 (70.5)	2196 (40.3)	269 (55.2)	
Unknown	3333 (5.9)	1808 (6.1)	746 (5.4)	464 (6.4)	249 (4.6)	66 (13.6)	
Income							<0.001
<$50,000	8133 (14.4)	5117 (17.4)	878 (6.4)	1942 (26.7)	96 (1.8)	100 (20.5)	
$50,000–$60,000	8507 (15.0)	5066 (17.2)	1707 (12.5)	1286 (17.7)	347 (6.4)	101 (20.7)	
$60,000–$70,000	19,711 (35.0)	8448 (28.7)	6829 (49.9)	2308 (31.7)	2024 (37.1)	102 (20.9)	
>$7,0000	20,012 (35.5)	10,842 (36.8)	4269 (31.2)	1737 (23.9)	2983 (54.7)	181 (37.2)	
Unknown	11 (0.019)	0 (0.0)	8 (0.1)	0 (0.0)	0 (0.0)	3 (0.6)	
Rural-urban Continuum code							<0.001
Metro areas (>1 million)	34,070 (60.4)	15,553 (52.8)	9899 (72.3)	4387 (60.3)	4096 (75.2)	135 (27.7)	
Metro areas (250,000–1 million)	11,200 (19.9)	6061 (20.6)	2622 (19.2)	1404 (19.3)	1021 (18.7)	92 (18.9)	
Metro areas (<250,000)	4355 (7.7)	2813 (9.5)	712 (5.2)	632 (8.7)	140 (2.6)	58 (11.9)	
Nonmetropolitan areas	6651 (11.8)	5046 (17.1)	450 (3.3)	850 (11.7)	193 (3.5)	112 (23.0)	
Unknown	98 (0.2)	0 (0.0)	8 (0.1)	0 (0.0)	0 (0.0)	90 (18.5)	
Region							<0.001
West	31,700 (56.2)	13,644 (46.3)	11,160 (81.5)	1700 (23.4)	4751 (87.2)	445 (91.4)	
South	14,161 (25.1)	9135 (31.0)	715 (5.2)	4028 (55.4)	261 (4.8)	22 (4.5)	
Northeast	8675 (15.4)	5046 (17.1)	1725 (12.6)	1494 (20.5)	401 (7.4)	9 (1.8)	
Mid-west	1838 (3.3)	1648 (5.6)	91 (0.7)	51 (0.7)	37 (0.7)	11 (2.3)	
SEER summary Stage							<0.001
Localized	25,674 (45.5)	13,989 (47.5)	6405 (46.8)	2656 (36.5)	2405 (44.1)	219 (45.0)	
Regional	20,246 (35.9)	9894 (33.6)	5057 (37.1)	2972 (40.9)	2127 (29.0)	178 (36.6)	
Distant	7922 (14.1)	4295 (14.6)	1615 (11.8)	1239 (17.0)	707 (13.0)	66 (13.6)	
Unknown	2532 (4.5)	1295 (4.4)	596 (4.4)	406 (5.6)	211 (3.9)	24 (4.9)	
Histologic subtype							<0.001
SCC	37,398 (66.3)	18,710 (63.5)	9334 (68.2)	5493 (75.5)	3529 (64.8)	332 (68.2)	
Adenocarcinoma	11,846 (21.0)	6987 (23.7)	2733 (20.0)	847 (11.6)	1187 (21.8)	92 (18.9)	
Others	3962 (7.1)	2089 (7.1)	940 (6.9)	445 (6.1)	455 (8.3)	33 (6.8)	
Unclassified	3168 (5.6)	1687 (5.7)	684 (5.0)	488 (6.7)	279 (5.1)	30 (6.2)	
Grade							<0.001
I	5303 (9.4)	3076 (10.4)	1274 (9.3)	400 (5.5)	498 (9.1)	55 (11.3)	
II	16,184 (28.7)	8529 (28.9)	3953 (28.9)	2049 (28.2)	1517 (27.8)	136 (27.9)	
III	15,575 (27.6)	7766 (26.3)	3897 (28.5)	2266 (31.2)	1532 (28.1)	114 (23.4)	
IV	1213 (2.2)	656 (2.2)	270 (2.0)	155 (2.1)	122 (2.2)	10 (2.1)	
Unknown	18,099 (32.1)	9446 (32.0)	4297 (31.4)	2403 (33.0)	1781 (32.7)	172 (35.3)	
Surgery							<0.001
No	24,114 (42.8)	11,889 (40.3)	5650 (41.3)	4066 (55.9)	2297 (42.1)	212 (43.5)	
Yes	31,637 (56.1)	17,242 (58.5)	7902 (57.7)	3116 (42.8)	3104 (57.0)	273 (56.1)	
Unknown	623 (1.1)	342 (1.2)	139 (1.0)	91 (1.3)	49 (0.9)	2 (0.4)	
Radiation therapy							<0.001
No/Unknown	26,517 (47.0)	14,452 (49.0)	6411 (46.8)	2863 (39.4)	2569 (47.1)	222 (45.6)	
Yes	29,857 (53.0)	15,021 (51.0)	7280 (53.2)	4410 (60.6)	2881 (52.9)	265 (54.4)	
Chemotherapy							<0.001
No/Unknown	30,592 (54.3)	16,450(55.8)	7338(53.6)	3624(49.8)	2930(53.8)	250(51.3)	
Yes	25,782 (45.7)	13,023 (44.2)	6353 (46.4)	3649 (50.2)	2520 (46.2)	237 (48.7)	

Abbreviations: IQR, inter quartile range; SCC, Squamous cell carcinoma.

## Data Availability

The original data presented in the study are openly available in the SEER database at https://seer.cancer.gov/data/.

## Supplementary Materials

The following supporting information can be downloaded at: https://www.mdpi.com/article/10.3390/healthcare13090964/s1, Figure S1: The path diagram demonstrating the socioeconomic and clinical factors mediate ethnic disparities in cervical cancer prognosis; Figure S2: Cumulative incidence of cervical cancer-specific and other cause of death in patients with cervical cancer; Figure S3: Cumulative incidence of cervical cancer-specific and other cause of death in patients with cervical cancer by age groups; Figure S4: Cumulative incidence of cervical cancer-specific and other cause of death in patients with cervical cancer by marital status; Figure S5: Cumulative incidence of cervical cancer-specific and other cause of death in patients with cervical cancer by periods of diagnosis; Figure S6: Cumulative incidence of cervical cancer-specific and other cause of death in patients with cervical cancer by income levels; Figure S7: Cumulative incidence of cervical cancer-specific and other cause of death in patients with cervical cancer by rurality; Figure S8: Cumulative incidence of cervical cancer-specific and other cause of death in patients with cervical cancer by regions; Figure S9: Cumulative incidence of cervical cancer-specific and other cause of death in patients with cervical cancer by grade; Figure S10: Cumulative incidence of cervical cancer-specific and other cause of death in patients with cervical cancer by stages; Figure S11: Cumulative incidence of cervical cancer-specific and other cause of death in patients with cervical cancer by histologic subtypes; Figure S12: The association between ethnicities and 5-year mortality among cervical cancer patients by different stages and surgery; Figure S13. The association between ethnicities and 5-year mortality among cervical cancer patients by different stages and histological types; Table S1: Cumulative mortality rate of all-cause death and cervical cancer-specific death for patients of cervical cancer overall and by ethnicity; Table S2: The associations between ethnicity, socioeconomic, clinical factors and 5-year mortality in patients with cervical cancer; Table S3: Hazard ratios of different ethnicity for 5-year mortality of all causes and cervical cancer-specific causes adjusted by incremental covariates; Table S4: Mediation analysis for the ethnic disparity in 5-year mortality from all cause and cervical cancer; Table S5: Mediation analysis for the ethnic disparity in 5-year mortality from all cause in different income groups; Table S6: The association between ethnicities and 5-year cervical cancer specific mortality among cervical cancer patients by different stages and surgery types; Table S7: The association between ethnicities and 5-year cervical cancer specific mortality among cervical cancer patients by different stages and histological types; Table S8: The association between ethnicities and 5-year cervical cancer specific mortality among cervical cancer patients by different stages and surgery types.
