# Supplementary material for: Mediation of Ethnic Disparity in the 5-Year Mortality of Cervical Cancer Patients in the US, 2001–2019
Source: Healthcare (Basel). 2025 Apr 22;13(9):964. doi: 10.3390/healthcare13090964 (PMC12071876; doi:10.3390/healthcare13090964)
Supplement: Supplementary file 1 [file healthcare-13-00964-s001.zip › healthcare-3464961-supplementary.pdf]

Supplementary Online Content

Table S1. Cumulative mortality rate of all-cause death and cervical cancer-specific death for patients of cervical cancer overall and by ethnicity.
Table S2. The associations between ethnicity, socioeconomic, clinical factors and 5-year mortality in patients with cervical cancer.
Table S3. Hazard ratios of different ethnicity for 5-year mortality of all causes and cervical cancer-specific causes adjusted by incremental covariates.
Table S4. Mediation analysis for the ethnic disparity in 5-year mortality from all cause and cervical cancer.
Table S5. Mediation analysis for the ethnic disparity in 5-year mortality from all cause in different income groups
Table S6. The association between ethnicities and 5-year cervical cancer specific mortality among cervical cancer patients by different stages and surgery types.
Table S7. The association between ethnicities and 5-year cervical cancer specific mortality among cervical cancer patients by different stages and histological types.
Table S8. The association between ethnicities and 5-year cervical cancer specific mortality among cervical cancer patients by different stages and surgery types.

Figure S1: The path diagram demonstrating the socioeconomic and clinical factors mediate ethnic disparities in cervical cancer prognosis.
Figure S2. Cumulative incidence of cervical cancer-specific and other cause of death in patients with cervical cancer.
Figure S3. Cumulative incidence of cervical cancer-specific and other cause of death in patients with cervical cancer by age groups
Figure S4. Cumulative incidence of cervical cancer-specific and other cause of death in patients with cervical cancer by marital status
Figure S5. Cumulative incidence of cervical cancer-specific and other cause of death in patients with cervical cancer by periods of diagnosis
Figure S6. Cumulative incidence of cervical cancer-specific and other cause of death in patients with cervical cancer by income levels.
Figure S7. Cumulative incidence of cervical cancer-specific and other cause of death in patients with cervical cancer by rurality.
Figure S8. Cumulative incidence of cervical cancer-specific and other cause of death in patients with cervical cancer by regions.
Figure S9. Cumulative incidence of cervical cancer-specific and other cause of death in patients with cervical cancer by grade.
Figure S10. Cumulative incidence of cervical cancer-specific and other cause of death in patients with cervical cancer by stages.
Figure S11. Cumulative incidence of cervical cancer-specific and other cause of death in patients with cervical cancer by histologic subtypes
Figure S12. The association between ethnicities and 5-year mortality among cervical cancer patients by different stages and surgery

types.

Figure S13. The association between ethnicities and 5-year mortality among cervical cancer patients by different stages and histological types

This supplementary material has been provided by the authors to give readers additional information about their work.

Table S1. Cumulative mortality rate of all-cause death and cervical cancer-specific death for patients of cervical cancer overall and by ethnicity.

Ethnicity	Cause of death	Cumulative death rate %						
		12m	24m	36m	48m	60m	120m	240m
Overall (N=56374)	All causes	15.0	23.1	27.2	29.4	31.0	34.4	36.3
	Cervical cancer specific death	13.1	20.3	23.7	25.4	26.5	28.3	28.8
Non-Hispanic White (N=29473)	All causes	15.8	23.7	27.7	30.1	31.6	35.3	37.3
	Cervical cancer specific death	13.9	20.9	24.3	26.1	27.2	29.1	29.5
Hispanic (All races) (N=13691)	All causes	10.6	17.9	21.7	23.5	24.8	27.4	28.9
	Cervical cancer specific death	9.2	15.8	18.9	20.4	21.4	22.9	23.4
Non-Hispanic Black (N=7273)	All causes	21.9	32.9	37.9	40.5	42.3	46.5	49.0
	Cervical cancer specific death	18.9	28.4	32.4	34.3	35.5	37.3	38.0
Asian or Pacific Islander (N=5450)	All causes	12.1	19.9	23.7	25.7	27.6	30.7	32.4
	Cervical cancer specific death	10.6	17.5	20.6	22.2	23.6	25.3	25.7
American Indian/Alaska Native (N=487)	All causes	14.4	24.6	29.0	31.0	32.7	35.3	35.9
	Cervical cancer specific death	12.9	21.6	25.7	26.5	27.9	29.8	29.8

Table S2. The associations between ethnicity, socioeconomic, clinical factors and 5-year mortality in patients with cervical cancer.

	Univariable Model for OS		Multivariable Model for all-cause mortality		Multivariable Model for cervical cancer specific mortality	
Characteristics	HR (95%CI) *	*P*	HR (95%CI) #	*P*	HR (95%CI) $	*P*
Ethnic						
NHW	1.00 (Reference)		1.00 (Reference)		1.00 (Reference)	
Hispanic (All races)	**0.81 (0.78;0.85)**	<.001	**0.83 (0.80, 0.87)**	<.001	**0.82 (0.78; 0.86)**	<.001
NHB	**1.49 (1.43;1.55)**	<.001	**1.09 (1.05, 1.14)**	<.001	**1.07 (1.02; 1.13)**	.007
API	**0.88 (0.84;0.93)**	<.001	**0.80 (0.76, 0.85)**	<.001	**0.83 (0.78; 0.88)**	<.001
AI/AN	1.03 (0.88;1.20)	.71	1.06 (0.89, 1.26)	.51	1.08 (0.88; 1.32)	.48
Age, per 1-y increase	**1.04 (1.04;1.04)**	<.001	**1.02 (1.02, 1.02)**	<.001	**1.01 (1.01; 1.01)**	<.001
Year of diagnosis						
2000-2004	1.00 (Reference)		1.00 (Reference)		1.0 (Reference)	
2005-2009	1.01 (0.97;1.05)	.78	**0.91 (0.87, 0.95)**	<.001	**0.93 (0.89; 0.98)**	.004
2010-2014	**1.06 (1.02;1.10)**	.004	**0.87 (0.84, 0.91)**	<.001	**0.90 (0.86; 0.94)**	<.001
2015-2019	0.97 (0.93;1.02)	.26	**0.78(0.75, 0.82)**	<.001	**0.78 (0.74; 0.82)**	<.001
Marital Status						
Married	1.00 (Reference)		1.00 (Reference)		1.00 (Reference)	
Unmarried	**1.69 (1.63;1.74)**	<.001	**1.21 (1.17, 1.25)**	<.001	**1.18 (1.13; 1.22)**	<.001
Unknown	**1.28 (1.20;1.37)**	<.001	1.00 (0.93, 1.07)	.99	1.02 (0.94; 1.11)	.69
Income						
＜$ 50,000	1.00 (Reference)		1.00 (Reference)		1.00 (Reference)	
$ 50,000- $ 60,000	**0.90 (0.86;0.95)**	<.001	1.03 (0.97, 1.09)	.33	1.05 (0.98; 1.12)	.19
$ 60,000- $ 70,000	**0.85 (0.81; 0.89)**	<.001	0.99 (0.92, 1.05)	.66	1.01 (0.94; 1.10)	.73
＞$ 7,0000	**0.73 (0.70;0.76)**	<.001	**0.91 (0.85, 0.98)**	<.001	0.93 (0.86; 1.01)	.08
Unknown	1.43 (0.54;3.81)	.48	1.72 (0.60, 4.94)	.32	2.12 (0.52; 8.63)	.29
Rural-urban Continuum code						
Metro areas (> 1million)	1.00 (Reference)		1.00 (Reference)		1.00 (Reference)	
Metro areas (250,000-1million)	1.03 (0.99;1.07)	.11	1.03 (0.98, 1.07)	.23	1.01 (0.96; 1.06)	.66

Metro areas (< 250,000)	**1.08 (1.02;1.14)**	<.001	1.00 (0.94, 1.07)	.99	1.02 (0.95; 1.10)	.57
Nonmetropolitan areas	**1.22 (1.17;1.28)**	<.001	1.03 (0.97, 1.10)	.35	1.01 (0.94; 1.09)	.74
Unknown	1.07 (0.76;1.52)	.69	1.18 (0.79, 1.78)	.42	0.89 (0.56; 1.43)	.63
Region						
West	1.00 (Reference)		1.00 (Reference)		1.00 (Reference)	
South	1.01 (0.97;1.05)	.78	1.03 (0.99, 1.08)	.18	0.99 (0.94; 1.05)	.74
Northeast	**1.06 (1.02;1.10)**	.004	0.96 (0.91, 1.00)	.05	**0.95 (0.90; 1.00)**	.05
Mid-west	0.97 (0.93;1.02)	.26	**0.86 (0.78, 0.94)**	<.001	**0.88 (0.79; 0.97)**	.01
Stage						
Localized	1.00 (Reference)		1.00 (Reference)		1.00 (Reference)	
Regional	**5.60 (5.34;5.87)**	<.001	**3.93 (3.71, 4.15)**	<.001	**4.56 (4.23; 4.91)**	<.001
Distant	**20.10 (19.1; 21.1)**	<.001	**11.91(11.23, 12.63)**	<.001	**13.61 (12.60; 14.69)**	<.001
Unknown	**6.99 (6.50; 7.51)**	<.001	**2.88 (2.66, 3.12)**	<.001	**3.53 (3.21; 3.88)**	<.001
Histologic subtype						
SCC	1.00 (Reference)		1.00 (Reference)		1.00 (Reference)	
Adenocarcinoma	**0.70 (0.67;0.73)**	<.001	**1.12 (1.07, 1.17)**	<.001	**1.14 (1.08; 1.19)**	<.001
Others	**1.19 (1.12;1.25)**	<.001	**1.44 (1.36, 1.52)**	<.001	**1.43 (1.34; 1.52)**	<.001
Unclassified	**2.36 (2.24;2.48)**	<.001	**1.63 (1.54, 1.72)**	<.001	**1.55 (1.45; 1.67)**	<.001
Grade						
I	1.00 (Reference)		1.00 (Reference)		1.00 (Reference)	
II	**2.38 (2.20;2.58)**	<.001	**1.45 (1.33, 1.58)**	<.001	**1.45 (1.33; 1.59)**	<.001
III	**4.16 (3.84;4.51)**	<.001	**1.88 (1.73, 2.04)**	<.001	**1.89 (1.73; 2.07)**	<.001
IV	**5.40 (4.84;6.02)**	<.001	**2.00 (1.79, 2.24)**	<.001	**2.03 (1.78; 2.31)**	<.001
Unknown	**2.80 (2.58;3.03)**	<.001	**1.39 (1.28, 1.51)**	<.001	**1.39 (1.27; 1.53)**	<.001
Surgery						
No	1.00 (Reference)		1.00 (Reference)		1.00 (Reference)	
Yes	**0.18 (0.17;0.18)**	<.001	**0.38 (0.36, 0.39)**	<.001	**0.43 (0.41; 0.46)**	<.001
Unknown	**0.86 (0.76;0.96)**	.008	0.96 (0.85, 1.08)	.50	1.05 (0.92; 1.21)	.45
Radiation therapy						
No/Unknown	1.00 (Reference)		1.00 (Reference)		1.00 (Reference)	
Yes	**2.13 (2.06;2.20)**	<.001	**0.79 (0.76, 0.83)**	<.001	**0.86 (0.81; 0.90)**	<.001

Chemotherapy						
No/Unknown	1.00 (Reference)		1.00 (Reference)		1.00 (Reference)	
Yes	**1.98 (1.92;2.04)**	<.001	**0.67 (0.64, 0.69)**	<.001	**0.79 (0.76; 0.83)**	<.001

Abbreviations: AI/AN, American Indian/Alaska Native; API, Asian or Pacific Islander; CI, confidence interval; HR, hazard ratio; NHB, Non-Hispanic Black; NHW, Non-Hispanic White; SEER, Surveillance, Epidemiology, and End Results Program; SCC, Squamous cell carcinoma.

* The HR and *P* values were calculated using cox regression models with no adjustment.

[#] The HR and *P* values for 5-year overall mortality were calculated using cox regression models adjusted by all other variates, including race/ethnics, age, marital status, years of diagnosis, income, rural-urban continuum code, regions, SEER summary stage, histologic subtype, grade, radiation therapy, chemotherapy and surgery.

[$] The HR and *P* values for 5-year cervical cancer specific mortality were calculated using Fine-Gray competing risk model adjusted by all other variables, including ethnics, age, marital status, years of diagnosis, income, rural-urban continuum code, regions, SEER summary stage, histologic subtype, grade, radiation therapy, chemotherapy and surgery.

Table S3. Hazard ratios of different ethnicity for 5-year mortality of all causes and cervical cancer-specific causes adjusted by incremental covariates.

	NHW	Hispanic (All races)		NHB		API		AI/AN	
Outcome		HR (95% CI)	*P*	HR (95% CI)	*P*	HR (95% CI)	*P*	HR (95% CI)	*P*
**All-cause mortality**									
Unadjusted	Reference	**0.81 (0.78,0.85)**	<.001	**1.49 (1.43,1.55)**	<.001	**0.88 (0.84,0.93)**	<.001	1.03 (0.88,1.20)	.71
+Age	Reference	**0.86 (0.83,0.90)**	<.001	**1.36 (1.30,1.41)**	<.001	**0.76 (0.72,0.80)**	<.001	**1.21 (1.03,1.41)**	**.01**
+Year of diagnosis	Reference	**0.86 (0.83,0.90)**	<.001	**1.36 (1.30,1.41)**	<.001	**0.76 (0.72,0.80)**	<.001	**1.21 (1.03,1.42)**	**.02**
+Marital Status	Reference	**0.85 (0.82,0.88)**	<.001	**1.27 (1.22,1.33)**	<.001	**0.78 (0.74,0.83)**	<.001	**1.17 (1.00,1.37)**	**.05**
+Income	Reference	**0.85 (0.82,0.89)**	<.001	**1.24 (1.19,1.30)**	<.001	**0.82 (0.78,0.87)**	<.001	1.16 (0.99,1.35)	.07
+Rural-urban	Reference	**0.85 (0.82,0.89)**	<.001	**1.25 (1.20,1.30)**	<.001	**0.82 (0.78,0.87)**	<.001	1.10 (0.93,1.31)	.27
+Regions	Reference	**0.85 (0.81,0.88)**	<.001	**1.25 (1.20,1.30)**	<.001	**0.82 (0.77,0.86)**	<.001	1.09 (0.91,1.29)	.35
+SEER summary	Reference	**0.85 (0.81,0.88)**	<.001	**1.17 (1.12,1.22)**	<.001	**0.83 (0.78,0.88)**	<.001	1.04 (0.88,1.24)	.63
+Histologic subtype	Reference	**0.85 (0.82,0.89)**	<.001	**1.16 (1.12,1.22)**	<.001	**0.83 (0.78,0.88)**	<.001	1.03 (0.86,1.22)	.77
+Grade	Reference	**0.85 (0.82,0.89)**	<.001	**1.16 (1.11,1.21)**	<.001	**0.83 (0.78,0.88)**	<.001	1.03 (0.87,1.23)	.71
+Surgery	Reference	**0.83 (0.79,0.86)**	<.001	**1.12 (1.07,1.17)**	<.001	**0.80 (0.76,0.85)**	<.001	1.03 (0.86,1.22)	.78
+Radiation therapy	Reference	**0.83 (0.80,0.87)**	<.001	**1.11 (1.07,1.16)**	<.001	**0.80 (0.76,0.85)**	<.001	1.05 (0.88,1.25)	.58
+Chemotherapy	Reference	**0.83 (0.80,0.87)**	<.001	**1.09 (1.05,1.14)**	<.001	**0.80 (0.76,0.85)**	<.001	1.06 (0.89,1.26)	.51
**Cervical cancer-specific**									
Unadjusted	Reference	**0.82 (0.78,0.85)**	<.001	**1.41 (1.35,1.48)**	<.001	**0.88 (0.83,0.93)**	<.001	1.02 (0.87,1.21)	.79
+Age	Reference	**0.86 (0.83,0.90)**	<.001	**1.31 (1.25,1.37)**	<.001	**0.79 (0.75,0.84)**	<.001	1.16 (0.98,1.37)	.09
+Year of diagnosis	Reference	**0.86 (0.83,0.90)**	<.001	**1.31 (1.25,1.37)**	<.001	**0.79 (0.75,0.84)**	<.001	1.16 (0.98,1.37)	.09
+Marital Status	Reference	**0.85 (0.81,0.89)**	<.001	**1.23 (1.18,1.29)**	<.001	**0.81 (0.77,0.86)**	<.001	1.13 (0.95,1.34)	.17
+Income	Reference	**0.85 (0.81,0.88)**	<.001	**1.21 (1.15,1.27)**	<.001	**0.84 (0.79,0.90)**	<.001	1.12 (0.95,1.33)	.19
+Rural-urban	Reference	**0.85 (0.81,0.89)**	<.001	**1.21 (1.16,1.27)**	<.001	**0.85 (0.80,0.90)**	<.001	1.11 (0.92,1.34)	.26
+Regions	Reference	**0.84 (0.80,0.87)**	<.001	**1.22 (1.16,1.28)**	<.001	**0.84 (0.79,0.89)**	<.001	1.09 (0.90,1.31)	.40
+SEER summary	Reference	**0.83 (0.79,0.87)**	<.001	**1.13 (1.07,1.19)**	<.001	**0.85 (0.80,0.90)**	<.001	1.05 (0.85,1.28)	.66
+Histologic subtype	Reference	**0.84 (0.80,0.88)**	<.001	**1.13 (1.08,1.19)**	<.001	**0.85 (0.80,0.90)**	<.001	1.04 (0.85,1.28)	.68
+Grade	Reference	**0.84 (0.80,0.88)**	<.001	**1.12 (1.07,1.18)**	<.001	**0.85 (0.80,0.90)**	<.001	1.05 (0.85,1.29)	.66
+Surgery	Reference	**0.82 (0.78,0.85)**	<.001	**1.09 (1.03,1.15)**	<.001	**0.83 (0.78,0.88)**	<.001	1.05 (0.86,1.30)	.62
+Radiation therapy	Reference	**0.82 (0.78,0.86)**	<.001	**1.08 (1.03,1.14)**	<.001	**0.83 (0.78,0.88)**	<.001	1.07 (0.87,1.31)	.51

+Chemotherapy	Reference	**0.82 (0.78,0.86)**	<.001	**1.07 (1.02,1.13)**	<.001	**0.83 (0.78,0.88)**	<.001	1.08 (0.88,1.32)	.48

Abbreviations: AI/AN, American Indian/Alaska Native; API, Asian or Pacific Islander; CI, confidence interval; HR, hazard ratio; NHB, Non-Hispanic Black; NHW, Non-Hispanic White; RE, relative effect; SEER, Surveillance, Epidemiology, and End Results Program.

[#] The HR and *P* values for 5-year overall mortality were calculated using cox regression models adjusted by covariates that were incrementally added (as showed in the table).

[$] The HR and *P* values for 5-year cervical cancer-specific mortality were calculated using Fine-Gray competing risk model adjusted by covariates that were incrementally added (as showed in the table).

Table S4. Mediation analysis for the ethnic disparity in 5-year mortality from all cause and cervical cancer.

	RE, % (95%CI) *		
**Mediators**	**NHB vs NHW**	**API vs NHW**	**Hispanic vs NHW**
**5-year mortality for all causes**			
Direct Effect	22.8 (15.0, 30.5)	102.8 (77.5, 133.6)	99.6(83.8,124)
Indirect Effect	77.2 (69.5, 85.0)	-2.8 (-33.6, 22.5)	0.4(-24.0,16.2)
**Indirect Effect Components**			
Marital status	7.1 (5.3, 8.6)	8.6 (4.7, 12.5)	-7.5 (-11.3, -4.4)
Income	1.7 (-0.1, 3.6)	13.3 (2.6, 23.3)	2.5 (-2.8, 9.9)
US Region	1.2 (-3.5, 3.6)	-3.7 (-14.6, 6.3)	1.4 (-7.0, 11.1)
Stage	29.6 (24.8, 34.3)	-17.0 (-38.1, -3.8)	9.0 (-0.3, 20.2)
Histologic subtypes	-0.2 (-1.5, 0.9)	3.7 (0.2, 6.8)	5.2 (3.2, 7.8)
Grade	5.9 (4.7, 7.2)	-7.4 (-13.2, -3.9)	-6.9 (-11.4, -3.2)
Surgery	26.7 (23.7, 30.3)	-4.1 (-17.1, 7.4)	-4.6 (-13.2, 2.4)
Radiation therapy	3.5 (1.9, 5.2)	-2.4 (-9.7, 3.5)	-0.5 (-7.4, 6.3)
Chemotherapy	5.6 (3.7, 8.2)	-2.1 (-7.7, 2.8)	-5.3 (-12.5, -1.4)
**5-year mortality for cervical cancer**			
Direct Effect	18.8 (10.1, 28.2)	118.9 (84.8, 164.4)	104.3 (83.5, 132.5)
Indirect Effect	81.2 (71.8, 89.9)	-18.9 (-64.4, 15.2)	-4.3 (-32.5, 16.5)
**Indirect Effect Components**			
Marital status	6.1 (4.6, 8.1)	8.6 (5.0, 15.5)	-5.9 (-8.3, -3.6)
Income	1.7 (0.3, 3.4)	14.5 (2.0, 27.2)	-0.3 (-6.4, 6.7)
US Region	0.7 (-1.5, 3.0)	-10.3 (-29.4, -0.5)	-5.6 (-20.2, 3.1)
Stage	34.2 (28.7, 40.1)	-23.4 (-59.1, -0.4)	11.3 (0.2, 23.9)
Histologic subtypes	-1.0 (-2.6, 0.6)	6.2 (0.3, 11.4)	6.5 (3.8, 10.8)
Grade	6.7 (5.6, 8.2)	-11.0 (-18.6, -4.2)	-7.1 (-14.7, -4.1)
Surgery	27.3 (22.5, 31.3)	-5.1 (-23.2, 5.5)	-2.9 (-10.8, 4.2)
Radiation therapy	4.0 (1.6, 6.2)	-0.6 (-10.7, 5.8)	-0.2 (-7.0, 4.3)
Chemotherapy	7.4 (5.1, 9.9)	-4.0 (-13.3, 2.8)	-6.1 (-13.1, -1.9)

Abbreviations: AI/AN, American Indian/Alaska Native; API, Asian or Pacific Islander; CI, confidence interval; NHB, Non-Hispanic Black; NHW, Non-Hispanic White; RE, relative effect; SEER, Surveillance, Epidemiology, and End Results Program.

*The RE value and its 95% confident interval was generated by generalized models with multiple additive regression tree using random seed of 10000.

Table S5. Mediation analysis for the ethnic disparity in 5-year mortality from all cause in different income groups

NHB vs NHW	RE, % (95%CI) *			
**Mediators**	**＜$ 50,000**	**$ 50,000- $ 60,000**	**$ 60,000- $ 70,000**	**＞$ 7,0000**
**5-year mortality for all causes**				
Direct Effect	21.9(6.2,35.8)	3.1(-31.9,31.1)	23.4(10.4,35.4)	35.3(25.3,50.5)
Indirect Effect	78.1(64.2,93.8)	96.9(68.9,131.9)	76.6(64.6,89.6)	64.7(49.5,74.7)
**Indirect Effect Components**				
Marital status	9.2(4.4,13.7)	6.7(1.2,12.5)	6.3(3.7,8.9)	4.4(1.9,6.8)
Rural-urban Continuum code	-2.1(-12.7,5.1)	3.9(-7.1,10.0)	0.01(-2.9,2.5)	-1.0(-2.2,0.0)
Stage	38.7(27.6,49.3)	47.4(36.8,55.2)	36.7(31.5,42.9)	22.9(17.1,29.4)
Histologic subtypes	-0.7(-5.3,3.8)	0.1(-3.1,3.1)	0.4(-1.9,2.8)	-2.7(-5.1,-0.7)
Grade	5.2(1.7,8.9)	6.2(3.5,9.3)	5.1(2.9,7.6)	7.0(4.4,10.4)
Surgery	29.3(21.9,36.8)	29.1(16.7,49.7)	26.9(23.7,31.2)	25.5(20.5,30.4)
Radiation therapy	2.4(-1.4,5.7)	4.1(-0.4,10.4)	3.9(1.3,7.4)	-1.1(-4.9,2.1)
Chemotherapy	1.8(-2.6,6.8)	7.6(3.5,13.5)	8.8(5.7,11.2)	4.0(1.3,6.8)

Abbreviations: CI, confidence interval; NHB, Non-Hispanic Black; NHW, Non-Hispanic White; RE, relative effect; SEER, Surveillance, Epidemiology, and End Results Program.

Table S6. The association between ethnicity and 5-year cervical cancer specific mortality among cervical cancer patients by different stages and surgery types.

Stage/ Surgery type	Hispanic vs NHW		NHB vs NHW		API vs NHW	
	HR (95%CI)	*P*	HR (95%CI)	*P*	HR (95%CI)	*P*
Localized						
Hysterectomy	1.07 (0.87,1.33)	.52	**1.55 (1.20,2.00)**	<.001	1.24 (0.94,1.62)	.12
Local destruction	0.99 (0.67,1.46)	.95	1.03 (0.68,1.57)	.88	1.00 (0.58,1.73)	>.99
No surgery	**0.80 (0.64,0.98)**	**.03**	**1.24 (1.02,1.52)**	**.04**	**0.60 (0.42,0.86)**	.004
Subtotal	0.92 (0.80,1.06)	.25	**1.34 (1.15,1.55)**	<.001	0.92 (0.75,1.12)	.40
Regional						
Hysterectomy	0.91 (0.78,1.06)	.24	**1.41 (1.17,1.71)**	<.001	1.00 (0.82,1.22)	>.99
Local destruction	**0.69 (0.52,0.92)**	**.01**	0.97 (0.74,1.26)	.79	0.76 (0.52,1.12)	.16
No surgery	**0.75 (0.70,0.81)**	<.001	0.99 (0.91,1.08)	.84	**0.74 (0.66,0.82)**	<.001
Subtotal	**0.78 (0.73,0.84)**	<.001	1.07 (0.99,1.15)	.08	**0.79 (0.72,0.86)**	<.001
Distant						
Hysterectomy	0.86 (0.67,1.10)	.22	1.21 (0.87,1.68)	.25	1.09 (0.79,1.51)	.59
Local destruction	0.84 (0.55,1.27)	.41	0.90 (0.61,1.33)	.58	1.17 (0.66,2.07)	.60
No surgery	**0.79 (0.72,0.86)**	<.001	0.98 (0.90,1.07)	.69	**0.83 (0.75,0.93)**	.001
Subtotal	**0.79 (0.73,0.86)**	<.001	1.00 (0.92,1.08)	.93	**0.87 (0.79,0.96)**	.007

Abbreviations: API, Asian or Pacific Islander; CI, confidence interval; HR, hazard ratio; NHB, Non-Hispanic Black; NHW, Non-Hispanic White; The HR and P values for 5-year cervical cancer specific mortality were calculated using Fine-Gray competing risk model adjusted by all other variables, including race/ethnics, age, marital status, years of diagnosis, income, rural-urban continuum code, regions, histologic subtype, grade, radiation therapy and chemotherapy.

Table S7. The association between ethnicity and 5-year cervical cancer specific mortality among cervical cancer patients by different stages and histological types.

Histologic subtyps/ Stage	Hispanic vs NHW		NHB vs NHW		API vs NHW	
	HR (95%CI)	*P*	HR (95%CI)	*P*	HR (95%CI)	*P*
**SCC**						
Localized	0.89 (0.75,1.06)	.20	**1.34 (1.13,1.60)**	<.001	0.92 (0.70,1.20)	.53
Regional	**0.78 (0.72,0.85)**	<.001	1.01 (0.93,1.09)	.89	**0.75 (0.68,0.84)**	<.001
Distant	**0.72 (0.66,0.80)**	<.001	0.95 (0.86,1.06)	.37	**0.81 (0.71,0.93)**	.002
**Subtotal**	**0.77 (0.73,0.82)**	<.001	1.03 (0.97,1.09)	.40	**0.76 (0.70,0.83)**	<.001
Adenocarcinomas						
Localized	1.09 (0.79,1.50)	.59	**1.63 (1.06,2.52)**	0.03	1.14 (0.76,1.71)	.53
Regional	0.88 (0.73,1.05)	.16	**1.58 (1.28,1.94)**	<.001	1.17 (0.94,1.45)	.16
Distant	0.88 (0.72,1.09)	.23	1.07 (0.87,1.32)	.53	0.98 (0.77,1.25)	.90
**Subtotal**	0.91 (0.80,1.04)	.18	**1.34 (1.16,1.55)**	<.001	1.13 (0.97,1.31)	.12
Others						
Localized	1.01 (0.65,1.57)	.95	1.05 (0.63,1.76)	.84	0.48 (0.24,0.95)	.04
Regional	**0.70 (0.55,0.89)**	.003	0.87 (0.65,1.16)	.33	0.82 (0.60,1.14)	.24
Distant	0.99 (0.77,1.27)	.93	1.15 (0.85,1.56)	.37	1.16 (0.87,1.55)	.32
**Subtotal**	0.87 (0.74,1.02)	.09	1.00 (0.83,1.21)	>.99	0.95 (0.78,1.16)	.61

Abbreviations: API, Asian or Pacific Islander; CI, confidence interval; HR, hazard ratio; NHB, Non-Hispanic Black; NHW, Non-Hispanic White; SCC, Squamous cell carcinomas; The HR and *P* values for 5-year cervical cancer specific mortality were calculated using Fine-Gray competing risk model adjusted by all other variables, including race/ethnics, age, marital status, years of diagnosis, income, rural-urban continuum code, regions, grade, radiation therapy, chemotherapy and surgery.

Table S8. The association between ethnicity and 5-year cervical cancer specific mortality among cervical cancer patients by different stages and surgery types.

Stage/ Surgery type	Histologic subtype	Hispanic vs NHW		NHB vs NHW		API vs NHW	
		HR (95%CI)	*P*	HR (95%CI)	*P*	HR (95%CI)	*P*
Local							
No surgery							
	SCC	**0.75 (0.58,0.97)**	.03	1.25 (1.00,1.57)	.05	0.66 (0.43,1.02)	.06
	Adenocarcinomas	1.13 (0.69,1.83)	.63	1.35 (0.72,2.52)	.34	0.69 (0.33,1.43)	.31
Local destruction							
	SCC	0.99 (0.61,1.60)	.96	1.29 (0.80,2.07)	.30	0.68 (0.32,1.45)	.32
	Adenocarcinomas	0.88 (0.33,2.32)	.79	0.20 (0.02,1.90)	.16	1.51 (0.64,3.56)	.35
Hysterectomy							
	SCC	1.09 (0.83,1.45)	.53	**1.47 (1.07,2.02)**	.02	1.37 (0.95,2.00)	.10
	Adenocarcinomas	1.28 (0.78,2.08)	.33	**2.70 (1.50,4.86)**	<.001	1.62 (0.95,2.76)	.08
Regional							
No surgery							
	SCC	**0.77 (0.70,0.84)**	<.001	0.94 (0.85,1.03)	.19	**0.73 (0.65,0.83)**	<.001
	Adenocarcinomas	0.81 (0.64,1.01)	.06	**1.56 (1.21,2.03)**	<.001	1.06 (0.81,1.39)	.69
Local destruction							
	SCC	**0.68 (0.49,0.94)**	.02	0.98 (0.73,1.31)	.87	0.70 (0.45,1.10)	.12
	Adenocarcinomas	0.79 (0.34,1.82)	.58	0.80 (0.33,1.89)	.60	1.72 (0.71,4.22)	.23
Hysterectomy							
	SCC	0.85 (0.69,1.04)	.11	**1.52 (1.20,1.94)**	<.001	0.88 (0.66,1.17)	.37
	Adenocarcinomas	1.10 (0.76,1.58)	.61	1.47 (0.96,2.27)	.08	1.24 (0.81,1.90)	.32
Distant							
No surgery							
	SCC	**0.72 (0.65,0.80)**	<.001	0.96 (0.86,1.07)	.41	**0.82 (0.71,0.95)**	.006
	Adenocarcinomas	0.89 (0.71,1.12)	.33	0.99 (0.79,1.24)	.92	0.87 (0.67,1.14)	.31
Local destruction							
	SCC	0.81 (0.49,1.35)	.42	0.76 (0.46,1.25)	.28	1.21 (0.53,2.79)	.65
	Adenocarcinomas	0.58 (0.17,1.94)	.37	2.34 (0.65,8.41)	.19	0.64 (0.01,31.88)	.82
Hysterectomy							
	SCC	**0.63 (0.42,0.93)**	.02	1.13 (0.74,1.72)	.58	0.67 (0.36,1.21)	.18
	Adenocarcinomas	1.00 (0.61,1.64)	>.99	1.93 (0.90,4.11)	.09	1.68 (0.85,3.30)	.13

Abbreviations: API, Asian or Pacific Islander; CI, confidence interval; HR, hazard ratio; NHB, Non-Hispanic Black; NHW, Non-Hispanic White; SCC, Squamous cell carcinomas; The HR and P values for 5-year cervical cancer specific mortality were calculated using Fine-Gray competing risk model adjusted by all other variables, including race/ethnics, age, marital status, years of diagnosis, income, rural-urban continuum code, regions, grade, radiation therapy and chemotherapy.

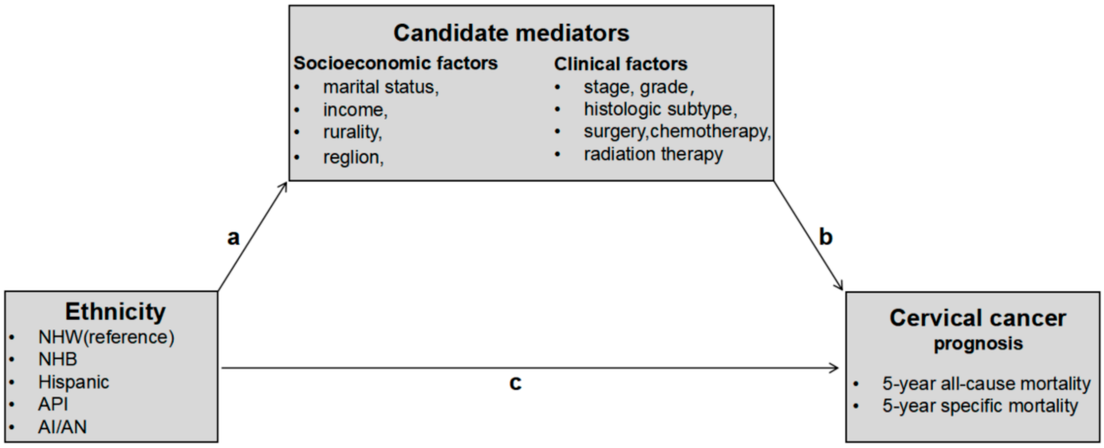

Figure S1: The path diagram demonstrating the socioeconomic and clinical factors mediate ethnic disparities in cervical cancer prognosis.

a indicates the direct effect of Ethnic on the Candidate mediators. **b** indicates the direct effect of Candidate mediators on the Cervical cancer prognosis. **c** indicates the direct effect of Ethnic on the cervical cancer prognosis, independent of the mediators. Abbreviations: AI/AN, American Indian/Alaska Native; API, Asian or Pacific Islander; NHW, Non-Hispanic White; NHB, Non-Hispanic Black.

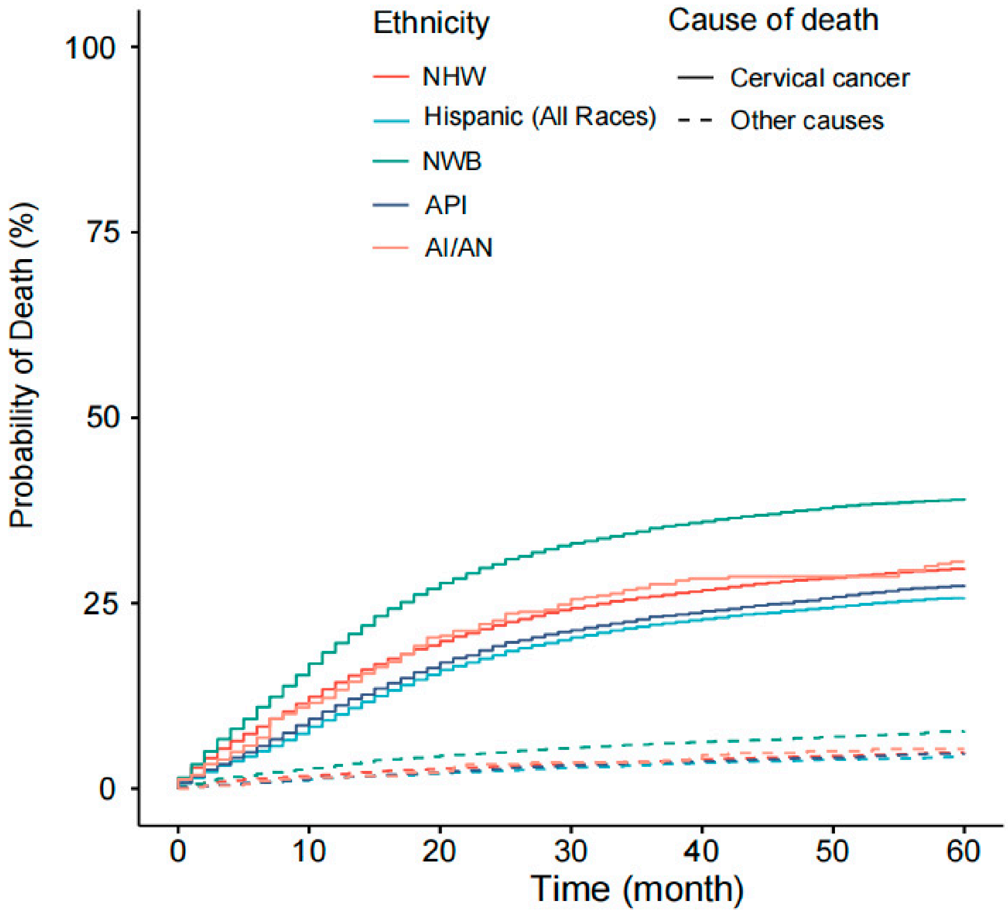

Figure S2. Cumulative incidence of cervical cancer-specific and other cause of death in patients with cervical cancer. Abbreviations: AI/AN, American Indian/Alaska Native; API, Asian or Pacific Islander; NHW, Non-Hispanic White; NHB, Non-Hispanic Black. The cumulative incidence was estimated using competing risk analysis in Fine and Gray's model.

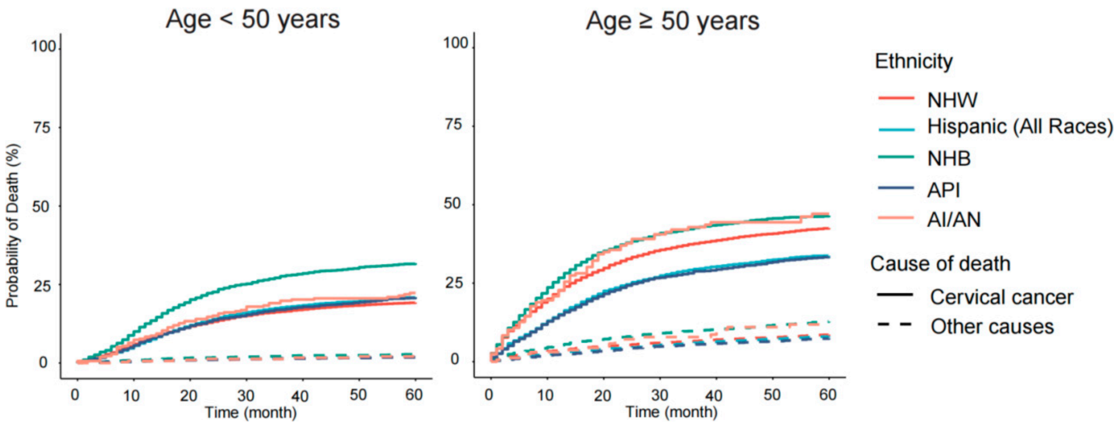

Figure S3. Cumulative incidence of cervical cancer-specific and other cause of death in patients with cervical cancer by age groups. Abbreviations: AI/AN, American Indian/Alaska Native; API, Asian or Pacific Islander; NHW, Non-Hispanic White; NHB, Non-Hispanic Black. The cumulative incidence was estimated using competing risk analysis in Fine and Gray's model.

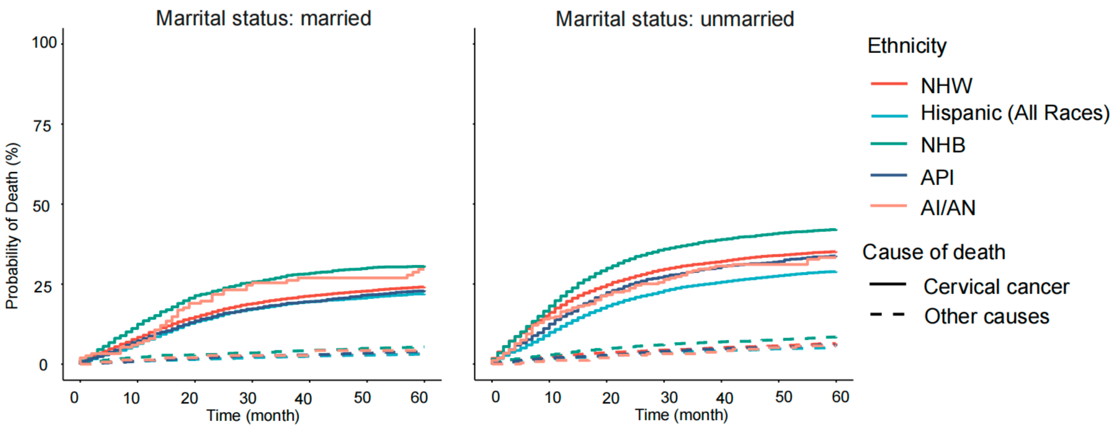

Figure S4. Cumulative incidence of cervical cancer-specific and other cause of death in patients with cervical cancer by marital status. Abbreviations: AI/AN, American Indian/Alaska Native; API, Asian or Pacific Islander; NHW, Non-Hispanic White; NHB, Non-Hispanic Black. The cumulative incidence was estimated using competing risk analysis in Fine and Gray's model.

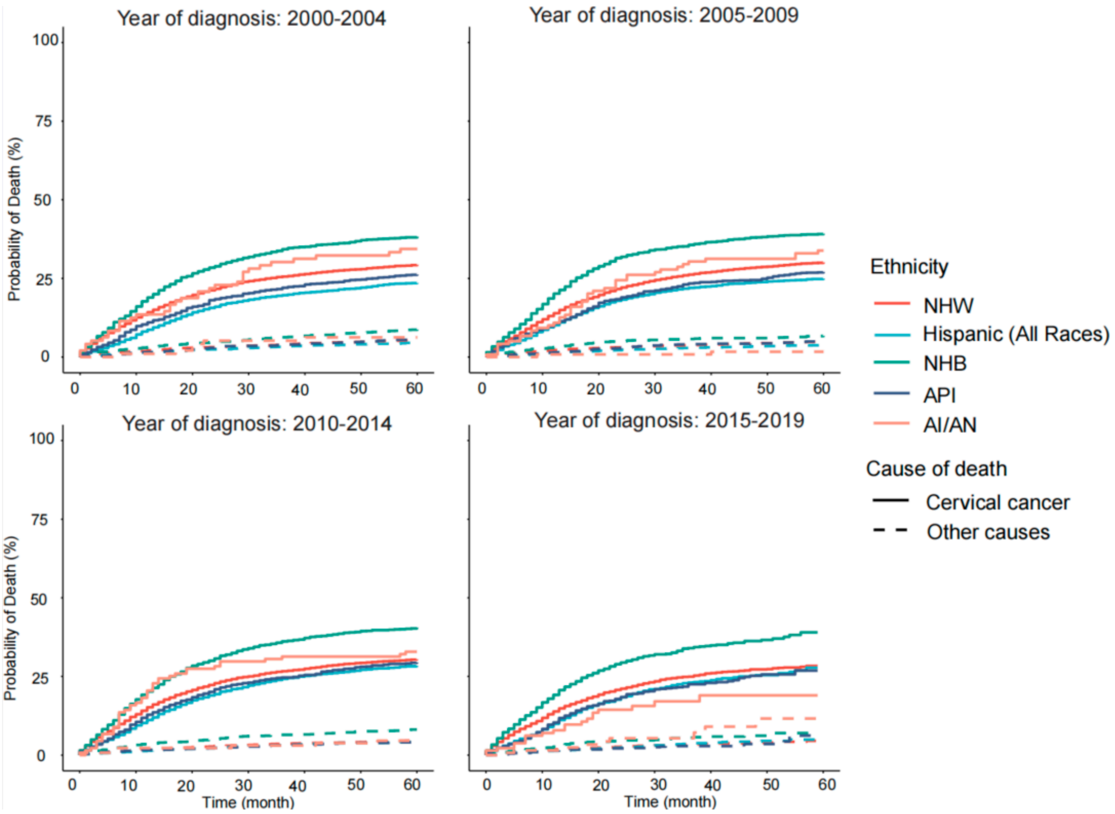

Figure S5. Cumulative incidence of cervical cancer-specific and other cause of death in patients with cervical cancer by periods of diagnosis. Abbreviations: AI/AN, American Indian/Alaska Native; API, Asian or Pacific Islander; NHW, Non-Hispanic White; NHB, Non-Hispanic Black. The cumulative incidence was estimated using competing risk analysis in Fine and Gray's model.

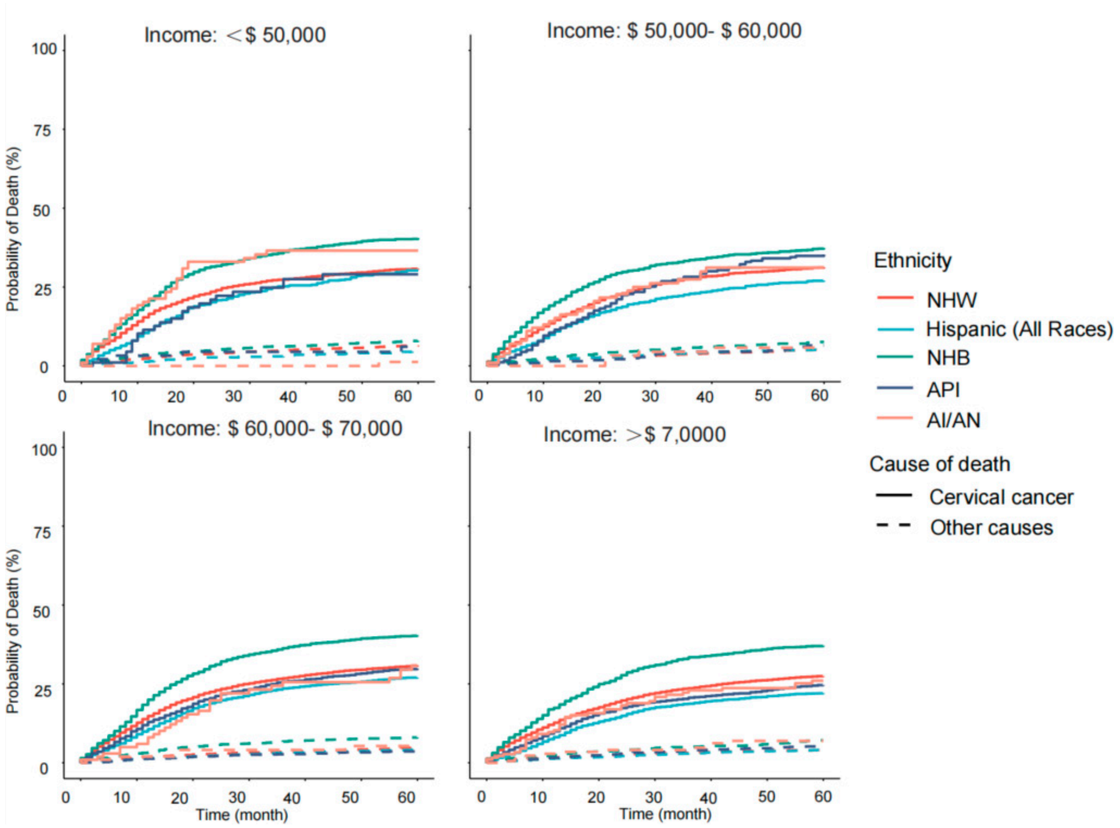

Figure S6. Cumulative incidence of cervical cancer-specific and other cause of death in patients with cervical cancer by income levels. Abbreviations: AI/AN, American Indian/Alaska Native; API, Asian or Pacific Islander; NHW, Non-Hispanic White; NHB, Non-Hispanic Black. The cumulative incidence was estimated using competing risk analysis in Fine and Gray's model.

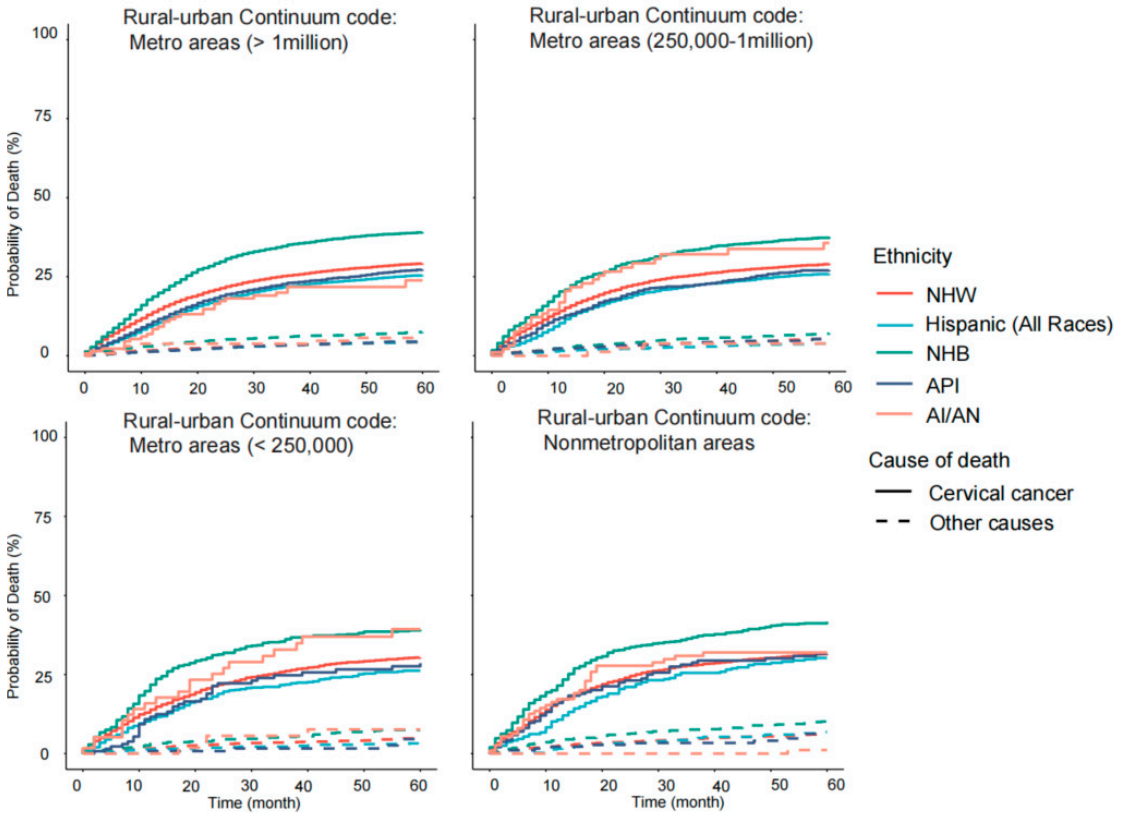

Figure S7. Cumulative incidence of cervical cancer-specific and other cause of death in patients with cervical cancer by rurality. Abbreviations: AI/AN, American Indian/Alaska Native; API, Asian or Pacific Islander; NHW, Non-Hispanic White; NHB, Non-Hispanic Black. The cumulative incidence was estimated using competing risk analysis in Fine and Gray’s model.

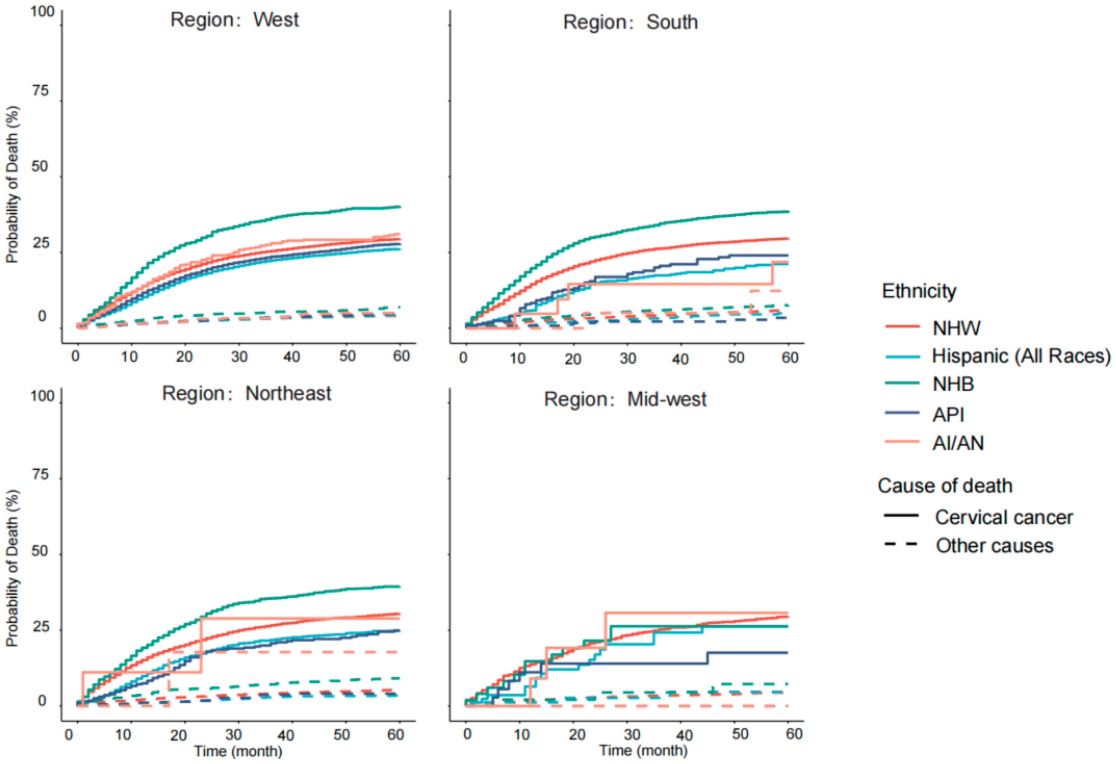

Figure S8. Cumulative incidence of cervical cancer-specific and other cause of death in patients with cervical cancer by regions. Abbreviations: AI/AN, American Indian/Alaska Native; API, Asian or Pacific Islander; NHW, Non-Hispanic White; NHB, Non-Hispanic Black. The cumulative incidence was estimated using competing risk analysis in Fine and Gray's model.

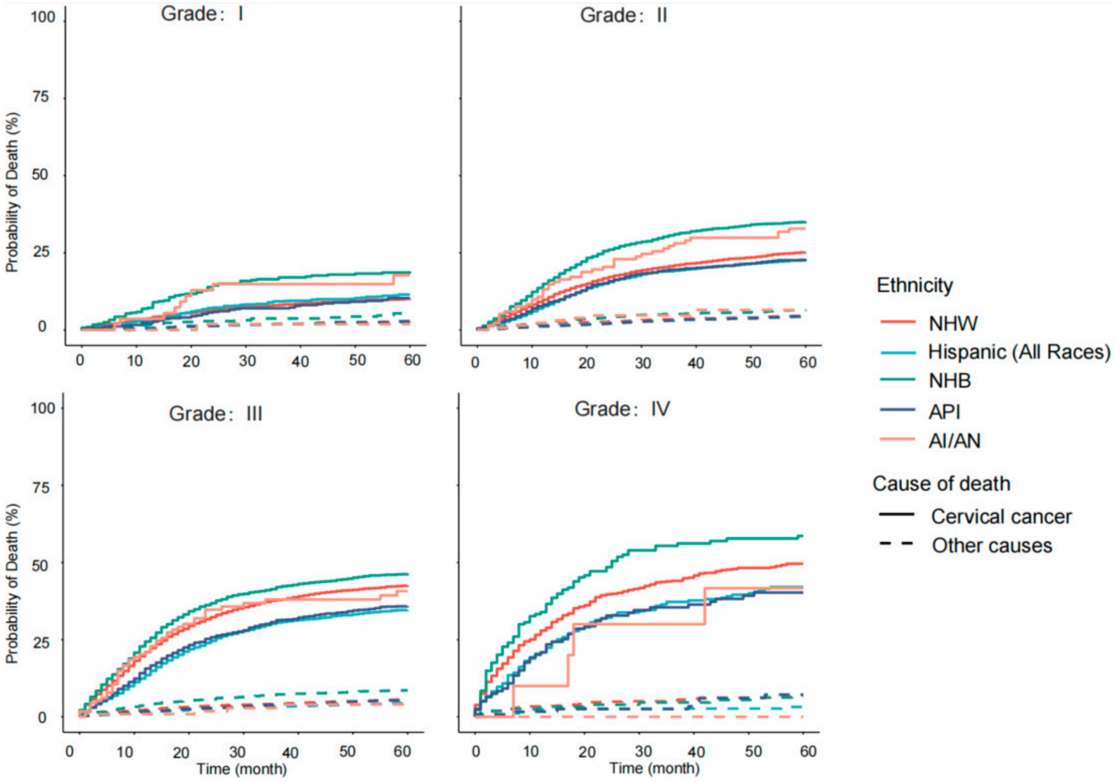

Figure S9. Cumulative incidence of cervical cancer-specific and other cause of death in patients with cervical cancer by grade. Abbreviations: AI/AN, American Indian/Alaska Native; API, Asian or Pacific Islander; NHW, Non-Hispanic White; NHB, Non-Hispanic Black. The cumulative incidence was estimated using competing risk analysis in Fine and Gray’s model.

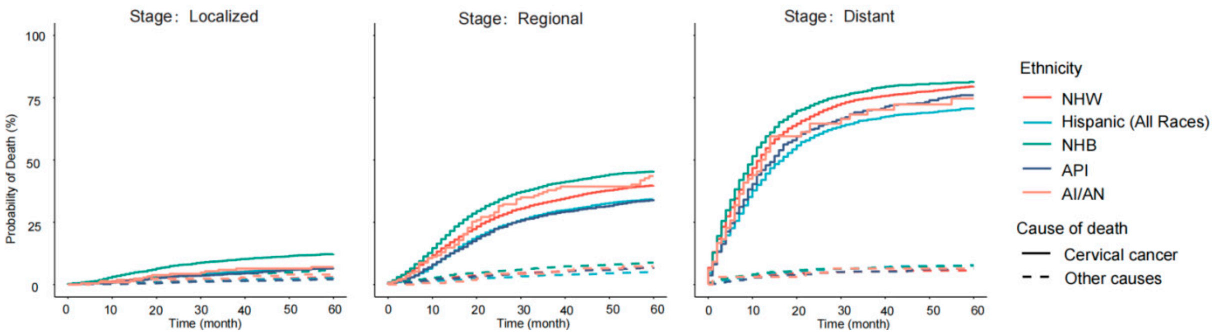

Figure S10. Cumulative incidence of cervical cancer-specific and other cause of death in patients with cervical cancer by stages. Abbreviations: AI/AN, American Indian/Alaska Native; API, Asian or Pacific Islander; NHW, Non-Hispanic White; NHB, Non-Hispanic Black. The cumulative incidence was estimated using competing risk analysis in Fine and Gray's model.

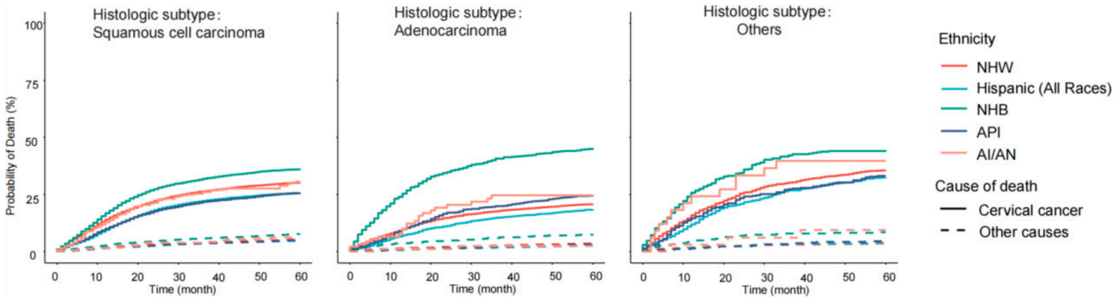

Figure S11. Cumulative incidence of cervical cancer-specific and other cause of death in patients with cervical cancer by histologic subtypes. Abbreviations: AI/AN, American Indian/Alaska Native; API, Asian or Pacific Islander; NHW, Non-Hispanic White; NHB, Non-Hispanic Black. The cumulative incidence was estimated using competing risk analysis in Fine and Gray's model.

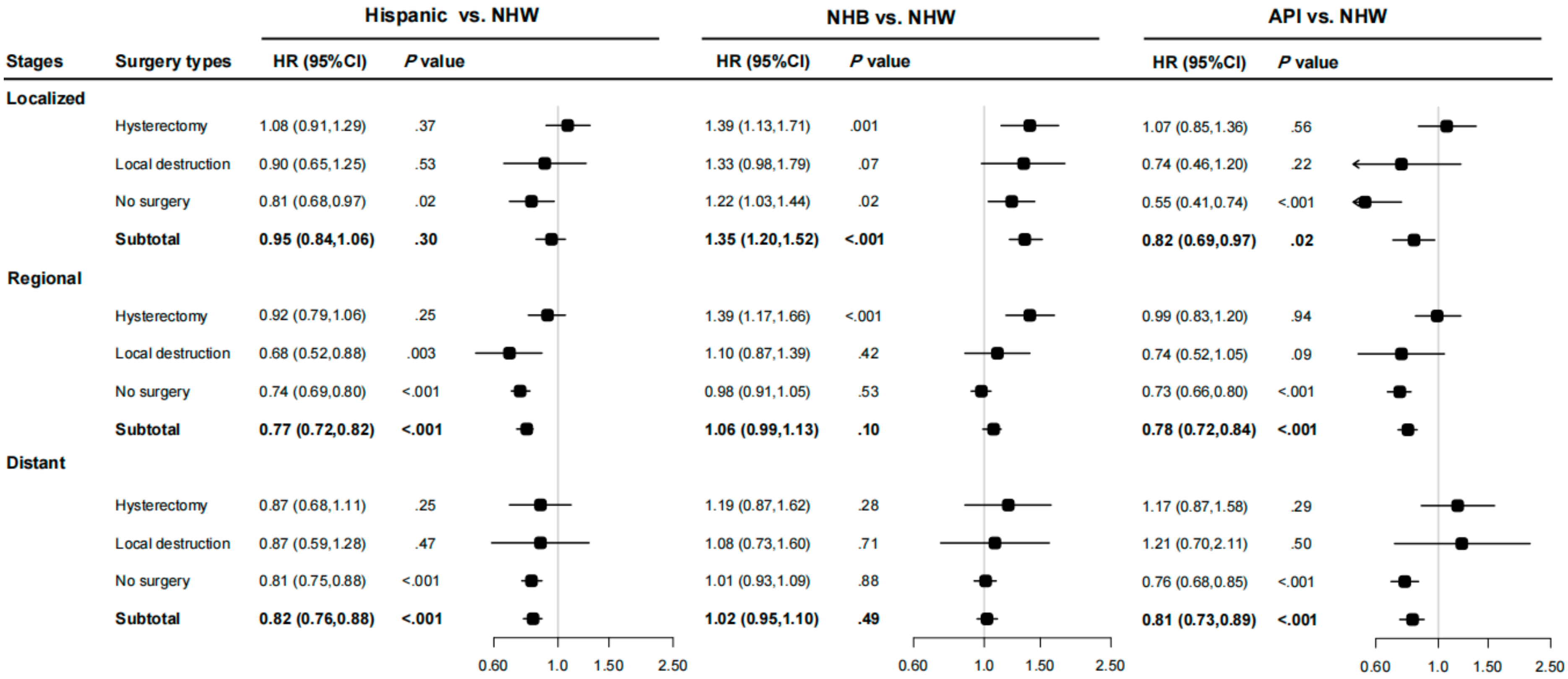

Figure S12. The association between ethnicities and 5-year mortality among cervical cancer patients by different stages and surgery types.

Abbreviations: API, Asian or Pacific Islander; CI, confidence interval; HR, hazard ratio; NHB, Non-Hispanic Black; NHW, Non-Hispanic White.

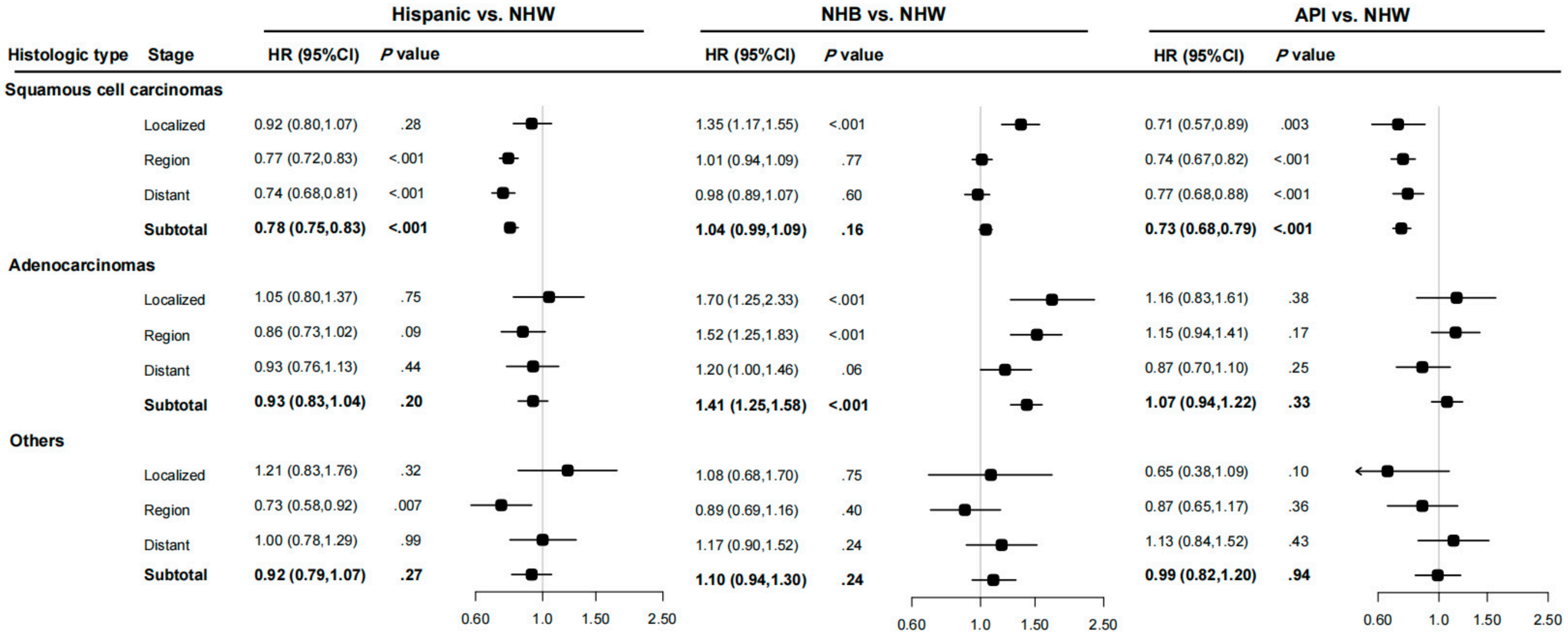

Histologic type	Stage	Hispanic vs. NHW HR (95%CI)	Hispanic vs. NHW *P* value	NHB vs. NHW HR (95%CI)	NHB vs. NHW *P* value	API vs. NHW HR (95%CI)	API vs. NHW *P* value
**Squamous cell carcinomas**							
	Localized	0.92 (0.80,1.07)	.28	1.35 (1.17,1.55)	<.001	0.71 (0.57,0.89)	.003
	Region	0.77 (0.72,0.83)	<.001	1.01 (0.94,1.09)	.77	0.74 (0.67,0.82)	<.001
	Distant	0.74 (0.68,0.81)	<.001	0.98 (0.89,1.07)	.60	0.77 (0.68,0.88)	<.001
	**Subtotal**	**0.78 (0.75,0.83)**	**<.001**	**1.04 (0.99,1.09)**	**.16**	**0.73 (0.68,0.79)**	**<.001**
**Adenocarcinomas**							
	Localized	1.05 (0.80,1.37)	.75	1.70 (1.25,2.33)	<.001	1.16 (0.83,1.61)	.38
	Region	0.86 (0.73,1.02)	.09	1.52 (1.25,1.83)	<.001	1.15 (0.94,1.41)	.17
	Distant	0.93 (0.76,1.13)	.44	1.20 (1.00,1.46)	.06	0.87 (0.70,1.10)	.25
	**Subtotal**	**0.93 (0.83,1.04)**	**.20**	**1.41 (1.25,1.58)**	**<.001**	**1.07 (0.94,1.22)**	**.33**
**Others**							
	Localized	1.21 (0.83,1.76)	.32	1.08 (0.68,1.70)	.75	0.65 (0.38,1.09)	.10
	Region	0.73 (0.58,0.92)	.007	0.89 (0.69,1.16)	.40	0.87 (0.65,1.17)	.36
	Distant	1.00 (0.78,1.29)	.99	1.17 (0.90,1.52)	.24	1.13 (0.84,1.52)	.43
	**Subtotal**	**0.92 (0.79,1.07)**	**.27**	**1.10 (0.94,1.30)**	**.24**	**0.99 (0.82,1.20)**	**.94**

Figure S13. The association between ethnicities and 5-year mortality among cervical cancer patients by different stages and histological types.

Abbreviations: API, Asian or Pacific Islander; CI, confidence interval; HR, hazard ratio; NHB, Non-Hispanic Black; NHW, Non-Hispanic White.
